# Sphingosine Kinase 2 Regulates Aryl Hydrocarbon Receptor Nuclear Translocation and Target Gene Activation

**DOI:** 10.1002/advs.202400794

**Published:** 2024-08-29

**Authors:** Shigetoshi Yokoyama, Imhoi Koo, Daisuke Aibara, Yuan Tian, Iain A. Murray, Stephanie L. Collins, Denise M. Coslo, Mari Kono, Jeffrey M. Peters, Richard L. Proia, Frank J. Gonzalez, Gary H. Perdew, Andrew D. Patterson

**Affiliations:** ^1^ Department of Veterinary and Biomedical Sciences Pennsylvania State University University Park PA 16802 USA; ^2^ Cancer Innovation Laboratory Center for Cancer Research National Cancer Institute National Institutes of Health Bethesda MD 20892 USA; ^3^ Department of Biochemistry and Molecular Biology Pennsylvania State University University Park PA 16802 USA; ^4^ Genetics and Biochemistry Branch National Institute of Diabetes and Digestive and Kidney Diseases National Institutes of Health Bethesda MD 20892 USA

**Keywords:** AHR, ceramide, sphingolipid, SPHK2

## Abstract

Sphingolipids play vital roles in metabolism and regulation. Previously, the aryl hydrocarbon receptor (AHR), a ligand‐activated transcription factor, was reported to directly regulate ceramide synthesis genes by binding to their promoters. Herein, sphingosine kinase 2 (SPHK2), responsible for producing sphingosine‐1‐phosphate (S1P), was found to interact with AHR through LXXLL motifs, influencing AHR nuclear localization. Through mutagenesis and co‐transfection studies, AHR activation and subsequent nuclear translocation was hindered by SPHK2 LXXLL mutants or SPHK2 lacking a nuclear localization signal (NLS). Similarly, an NLS‐deficient AHR mutant impaired SPHK2 nuclear translocation. Silencing SPHK2 reduced AHR expression and its target gene *CYP1A1*, while SPHK2 overexpression enhanced AHR activity. SPHK2 was found enriched on the *CYP1A1* promoter, underscoring its role in AHR target gene activation. Additionally, S1P rapidly increased AHR expression at both the mRNA and protein levels and promoted AHR recruitment to the *CYP1A1* promoter. Using mouse models, AHR deficiency compromised SPHK2 nuclear translocation, illustrating a critical interaction where SPHK2 facilitates AHR nuclear localization and supports a positive feedback loop between AHR and sphingolipid enzyme activity in the nucleus. These findings highlight a novel function of SPHK2 in regulating AHR activity and gene expression.

## Graphical abstract

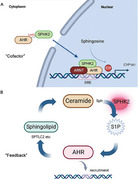

## 1 Introduction

Cellular metabolism is crucial for producing chemical components essential for cell proliferation and gene regulation events in the nucleus. Recent evidence suggests that subnetworks of key metabolic reactions, such as the tricarboxylic acid (TCA) cycle, occur within the nucleus.^[^ ^1^ ^]^ These nuclear metabolic pathways generate molecules that modify chromatin and alter gene expression, allowing the cell to rapidly respond to changing environmental conditions.

Sphingolipids are important components of cellular architecture and function as signaling molecules, which are important in diseases such as obesity and cancer, as well as key cellular responses like apoptosis.^[^ ^2^, ^3^ ^]^ Emerging data supports the concept that the activity of sphingolipids and their biosynthetic enzymes may not be limited to the endoplasmic reticulum and Golgi network but may also be active within the nucleus.^[^ ^4^, ^5^, ^6^, ^7^ ^]^ However, the precise mechanisms behind this nuclear role for sphingolipids are largely unknown.^[^ ^8^, ^9^ ^]^

The aryl hydrocarbon receptor (AHR) is a member of the basic‐helix‐loop‐helix/Per‐Arnt‐Sim (bHLH/PAS) gene family and is a ligand‐activated transcription factor that binds to chemicals such as 2,3,7,8‐tetrachlorodibenzo‐*p*‐dioxin (TCDD, commonly known as “dioxin”).^[^ ^10^ ^]^ In the absence of ligand, AHR resides in the cytoplasm with X‐associated protein 2 (XAP‐2), heat shock protein 90 (HSP90),^[^ ^11^ ^]^ and p23.^[^ ^12^ ^]^ Upon agonist binding, the AHR undergoes conformational changes and translocates into the nucleus where it forms a heterodimer with the aryl hydrocarbon receptor nuclear translocator (ARNT) capable of binding to dioxin response elements (DREs) of AHR target genes.^[^ ^10^ ^]^ One of the most extensively studied target genes of the AHR is the cytochrome P450 family 1 subfamily A member 1 (*CYP1A1*). Ligands for the AHR not only include xenobiotics such as TCDD, but also endogenous compounds including tryptophan derivatives.^[^ ^13^ ^]^ Importantly, the AHR modulates several pathways that regulate normal cellular homeostasis.^[^ ^13^ ^]^ For example, AHR activation has been linked to immune cell and barrier epithelial cell differentiation and cell lineage fate.^[^ ^14^, ^15^, ^16^, ^17^ ^]^ Perhaps the best example of the role of AHR in epithelial cell differentiation is through the terminal differentiation of the skin barrier, where the AHR plays a central role in keratinocyte maturation.^[^ ^18^ ^]^ The basic mechanisms that drive these events remain unclear and thus require additional investigation.

Previous studies have shown that the AHR regulates genes involved in ceramide synthesis, such as serine palmitoyltransferase long chain base subunit 2 (*Sptlc2)*, through direct binding to their promoter regions.^[^ ^19^, ^20^ ^]^ Additionally, ceramide synthesis enzymes were shown to influence transcription through direct binding to DNA, as observed with ceramide synthase Schlank in the model organism Drosophila melanogaster.^[^ ^21^ ^]^ Based on these findings, we investigated whether sphingolipids and their biosynthetic proteins may play a role in the regulation of AHR transcription.

Here, we report that the multifunctional lipid kinase, sphingosine kinase 2 (SPHK2) interacts with AHR through LXXLL (where L is leucine and X is any amino acid) consensus motifs. Point mutation of these motifs by site‐directed mutagenesis significantly reduced the binding of SPHK2 with AHR. Furthermore, AHR failed to translocate to the nucleus with co‐transfection of SPHK2 with either the nuclear localization signal (NLS) deletion or LXXLL motif mutation. These results suggest a new function of SPHK2 for regulating the translocation of the AHR into the nucleus. Silencing of SPHK2 through specific inhibitors or gene knockdown by RNAi or CRISPR/Cas9 leads to a decrease of AHR expression and signaling, while overexpression of SPHK2 increases AHR expression. Additionally, we found that sphingosine‐1‐phosphate (S1P), a product of SPHK2 phosphorylation, increases AHR expression. Furthermore, we show that SPHK2 is present on the *CYP1A1* promoter in a DRE‐enriched region likely in a complex with AHR. Silencing of SPHK2 reduces the recruitment of AHR to the *CYP1A1* promoter, while S1P administration rapidly enhances recruitment of AHR to DREs in the *CYP1A1* promoter, suggesting that SPHK2 may serve as a co‐regulator or stabilization factor for AHR‐mediated transcriptional activity. We further validate these findings from cell culture models using livers sections from wildtype and *Ahr*‐null mice treated with TCDF. Overall, our results suggest that SPHK2 has multiple roles, and functions as regulator of AHR translocation and establishes a positive feedback mechanism between sphingolipid synthesis proteins, S1P production, and the regulation of AHR and its target genes in the nucleus.

## 2 Results

### 2.1 Ceramide Synthesis Proteins have LXXLL Motifs

To identify ceramide synthesis proteins that may interact with the AHR and function as coregulators (coactivator/corepressors), we surveyed ceramide synthesis proteins for the presence of LXXLL motifs, which are known to be an important signature for nuclear receptor, coactivator, and corepressor interaction^[^ ^22^, ^23^, ^24^, ^25^, ^26^, ^27^, ^28^ ^]^ and identified numerous ceramide‐related proteins that contain this motif (Table S3, Supporting Information). Next, we compared human and mouse amino acid sequences for LXXLL motifs and focused on proteins in which the LXXLL motif was followed by a hydrophobic residue (isoleucine, I; valine, V; leucine, L; phenylalanine, F; cysteine, C; methionine, M; alanine, A; and, tryptophan, W) or basic amino acid (arginine, A; histidine, H; and, lysine, K) at the −1 position, which is reported to be important for nuclear receptor interaction.^[^ ^22^, ^23^ ^]^ Notably, we focused on candidates according to the following criteria: 1) −1 position is a hydrophobic or basic amino acid; 2) has multiple LXXLL motifs; and, 3) protein localization was reported to be in the nucleus or that we could confirm nuclear localization by immunohistochemical analysis or on the Human Protein atlas (https://www.proteinatlas.org/). In addition to many sphingolipid and ceramide synthesis proteins, we found that SPHK2, which produces sphingosine‐1‐phosphate (S1P) in cells, has multiple LXXLL motifs and is known to shuttle between cytoplasm and nucleus.^[^ ^29^, ^30^ ^]^ In contrast to SPHK2, another sphingosine kinase isoform which generates S1P, SPHK1 (sphingosine kinase 1) does not possess any LXXLL motifs (Table S3 and Figure S3, Supporting Information). We compared the protein interactions between these two sphingosine kinases and AHR by immunoprecipitation (IP) assays (Figure S3A,B, Supporting Information). Results demonstrated that SPHK2 (which contains multiple LXXLL motifs) strongly interacts with AHR while less interaction was found with SPHK1 (no LXXLL motifs). Thus, we focused on the relationship between AHR and SPHK2 in this study.

### 2.2 SPHK2 Interacts with AHR/ARNT Heterodimer

**Figure 1:**
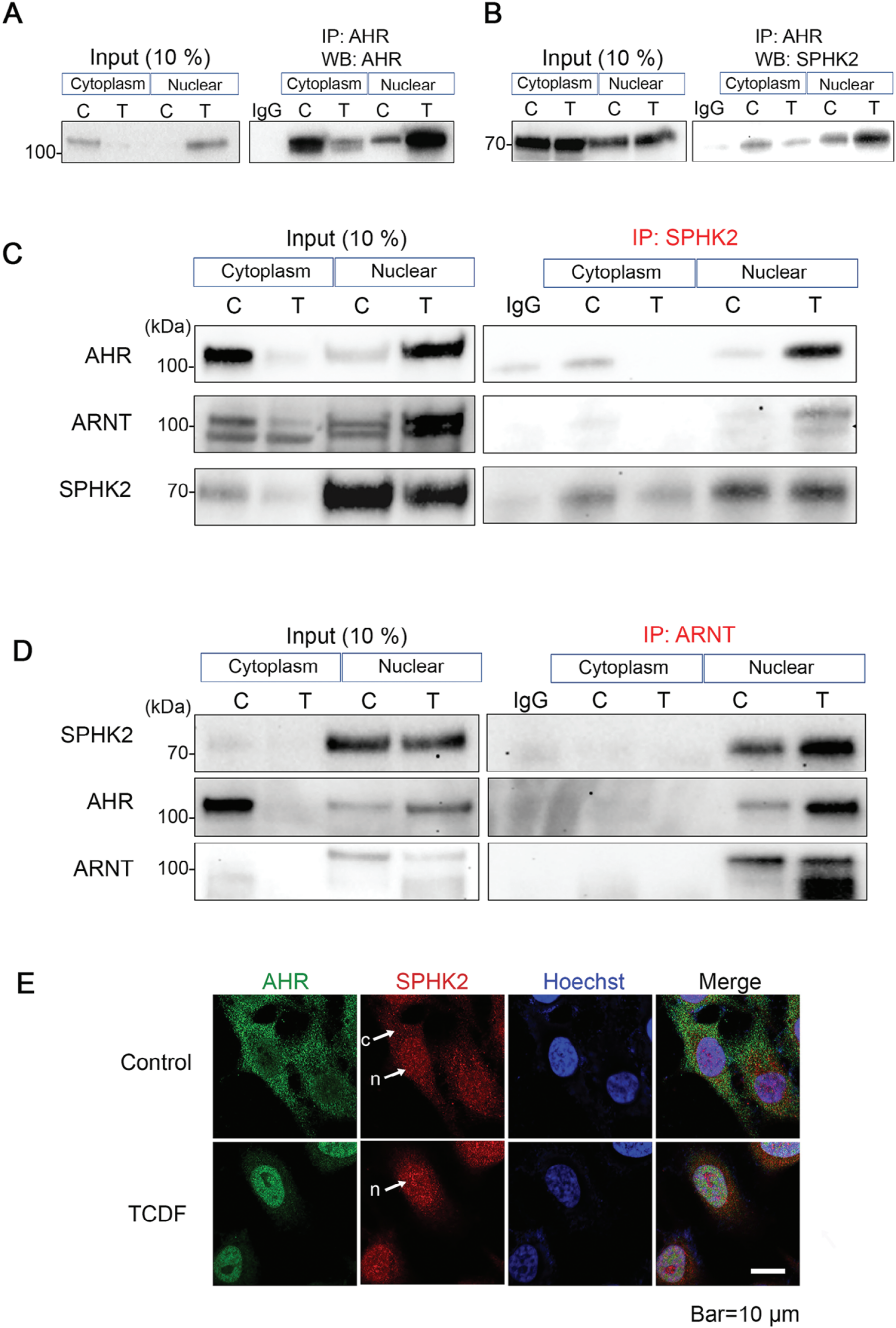
Endogenous AHR and SPHK2 interact in HeLa cells. A,B) HeLa cells treated with or without TCDF were subjected to cytoplasmic/nuclear extract fractionation and immunoprecipitated using AHR antibody and western blotting analysis was performed using an (A) AHR antibody or (B) SPHK2 antibody. C, Control; T, TCDF treated samples. C) After IP of SPHK2, western blotting analysis was performed with AHR, ARNT and SPHK2 antibodies. C, Control; T, TCDF treated samples. D) After IP of ARNT, western blotting analysis was performed with AHR, SPHK2 and ARNT antibodies. C, Control; T, TCDF treated samples. E) Immunofluorescent double staining using anti‐AHR and anti‐SPHK2 antibodies. The nucleus was counterstained with Hoechst 33 342. Arrows c indicate the cytoplasm, n indicate the nucleus. Bar is 10 µm. Data are representative images from three independent experiments.

To explore a potential interaction between AHR and SPHK2, HeLa cells were used as they express relatively high levels of AHR^[^ ^31^ ^]^ and CRISPR/Cas9 knockouts were available.^[^ ^32^ ^]^ Upon the addition of an agonist, the AHR translocates from the cytoplasm into the nucleus within 30 min (**Figure** **1**A). 2,3,7,8‐Tetrachlorodibenzofuran (TCDF) was utilized as a potent highly specific AHR agonist. IP using an AHR antibody confirmed that SPHK2 interacts with endogenous AHR (Figure 1B; Figure S3, Supporting Information). To determine if SPHK2 impacts the expression of AHR and its downstream target genes, SPHK2 IP experiments were performed. Indeed, we noted that SPHK2 co‐immunoprecipitates with AHR in nuclear extracts and this interaction is ligand‐dependent (Figure 1C). We also confirmed the interaction between AHR and SPHK2 using an additional cell line (Huh7, a human hepatoma cell line, (Figure S4A, Supporting Information) and a different AHR agonist, indolo[3,2‐b] carbazole (ICZ) (Figure S4B, Supporting Information). IP experiments also revealed that SPHK2 interacts with the AHR heterodimerization partner ARNT or the AHR/ARNT heterodimer after TCDF treatment (Figure 1C). ARNT IP further revealed the presence of an AHR/ARNT/SPHK2 complex (Figure 1D). Both SPHK2 and AHR interactions with ARNT were enhanced by TCDF treatment, suggesting that SPHK2 interacts with the AHR/ARNT heterodimer which is indispensable for downstream target gene activation by AHR. These results suggest a newly identified role for SPHK2 via interaction with the AHR/ARNT heterodimer.

Next, we examined the localization of SPHK2 in HeLa cells by immunofluorescence visualization (Figure 1E, see also Huh7 cells in Figure S5, Supporting Information). In untreated HeLa cells, SPHK2 is relatively uniformly expressed throughout the cell (red in upper panel, arrows), yet following TCDF treatment, SPHK2 is predominantly nuclear (Figure 1E, arrow n in the lower panel) and co‐localizes with AHR (Figure 1E, green). Collectively these results suggest that SPHK2 co‐localizes with AHR in the nucleus following TCDF treatment, showing similar nuclear translocation kinetics as the AHR.

### 2.3 SPHK2 Inhibition or Knockdown Reduces AHR Presence in Nuclear Extracts and Reduces AHR Target Gene Activation

**Figure 2:**
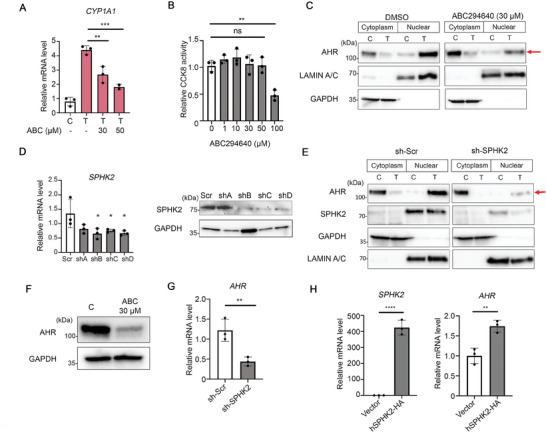
Knockdown of SPHK2 downregulates AHR expression. A) qRT‐PCR analysis of *CYP1A1* expression in HeLa cells after incubation with or without TCDF (0.1 µM) for 2 h or pretreatment with SPHK2 inhibitor (ABC, ABC294640) for 2 h. Carrier solvent DMSO was used as a control. B) CCK8 assay results after 2 h incubation with different doses of ABC294640 (µM). C) HeLa cells after treatment with 0.1 µM TCDF for 30 min or pretreatment with ABC294640 for 2 h followed by 0.1 µM TCDF for 30 min, cells were fractionated into cytoplasmic and nuclear extracts and subjected to western blotting analysis with the indicated antibodies. Red arrow indicates the reduced level of AHR protein in the ABC294640 + TCDF treated nucleus. LAMIN A/C antibody and GAPDH antibody were used for the loading control of nuclear and cytoplasmic extracts, respectively. n = 3 D) HeLa cells were transfected with *SPHK2* shRNA for 48 h and the efficiency of each shRNA plasmid knockdown was assessed by qRT‐PCR (left) and western blotting (right). n = 3 E) Western blot analysis of HeLa cells transfected with *SPHK2* shRNA or shRNA‐scramble (scr) for 48 h. Cells were treated 30 min with or without 0.1 µM TCDF, extracted, and proteins detected with the indicated antibodies. n = 3 F) ABC294640 incubation in HeLa cells for 2 h decreased AHR expression as determined by western blotting analysis. n = 3 G) shRNA for *SPHK2* for 48 h decreases *AHR* mRNA in HeLa cells. (H) Transient overexpression of human SPHK2 for 48 h and qRT‐PCR was performed. All data mean ± S.D. (n = 3). ^****^ *p* < 0.0001, ^***^ *p* < 0.001, ^**^ *p* < 0.01, ^*^ *p* < 0.05.

To further determine the role of SPHK2 in AHR signaling, the SPHK2 inhibitor ABC294640^[^ ^33^ ^]^ was utilized. ABC294640 decreased the expression of the AHR target gene *CYP1A1* following TCDF treatment (**Figure** **2**A). The cytotoxicity of ABC294640 was minimal and did not show inhibitory effects in HeLa cells until the maximal does of 100 µM was used (Figure 2B). Hence, a concentration of 30 µM ABC294640 was used in subsequent experiments.

To analyze the effects of ABC294640 on AHR translocation and nuclear retention following TCDF treatment, AHR protein levels were examined in cells treated with and without TCDF. In control cells (no ABC294640) with TCDF treatment, AHR was retained in nuclear extracts. In contrast, in TCDF and ABC294640‐treated HeLa cells, AHR levels in nuclear extracts were significantly reduced (Figure 2C, red arrow).

To confirm that the results obtained using ABC294640 were not due to off‐target effects, a small hairpin RNA (shRNA) silencing approach was used to reduce human *SPHK2* gene expression. Three of the four shRNA SPHK2 vectors (shB, shC, and shD) reduced both RNA and protein levels of SPHK2 (Figure 2D). Subcellular fractionation and western blot analysis revealed that using shRNA, the results were similar with the experiment using the ABC294640 inhibitor (Figure 2E). Knockdown of SPHK2 also significantly reduced AHR retention in nuclear extracts after TCDF exposure (Figure 2E, red arrow).

ABC294640 treatment also diminished AHR protein levels (Figure 2F). Silencing of SPHK2 by shRNA also decreased *AHR* at both the mRNA and AHR protein level (Figure 2G; Figure S6, Supporting Information). By contrast, forced expression of human *SPHK2* increased *AHR* gene expression (Figure 2H). Considering that SPHK2 is present in the nucleus, and that nuclear AHR retention was significantly affected by both ABC294640 and shRNA treatment, we concluded that SPHK2 plays a pivotal role in regulating AHR levels and AHR signaling.

### 2.4 S1P Rapidly Increases AHR and SPHK2 Expression

**Figure 3:**
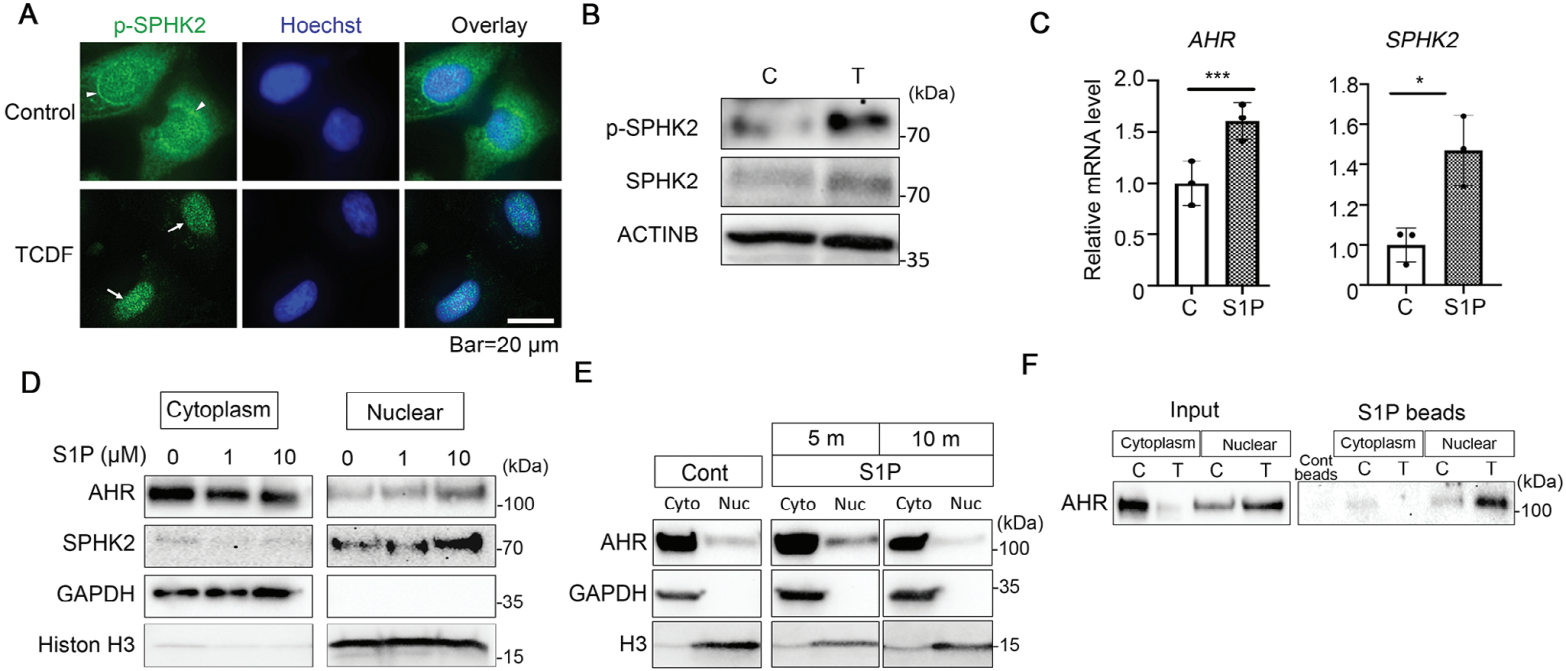
AHR agonist activates the SPHK2/S1P pathway resulting in rapid nuclear retention of the AHR and SPHK2. A) Immunofluorescent images of HeLa cells for p‐SPHK2 levels after TCDF (0.1 µM) incubation for 20 min. Arrowheads indicate the peri‐nuclear region staining of p‐SPHK2 in control cells and arrows indicate the nuclear expression of p‐SPHK2 in TCDF treated cells. Hoechst 33342 was used for the counter staining of nuclei. Bar = 20 µm. Data are representative images from three independent experiments. B) Western blot analysis of HeLa cells after TCDF treatment for 15 min. C, Control; T, TCDF (n = 3). C) qRT‐PCR analysis of HeLa cells for *AHR* and *SPHK2* mRNA levels after S1P incubation for 30 min. C: Control. Data are mean ± S.D. (n = 3). ^****^ *p* < 0.0001, ^*^ *p* < 0.05. D) Western blotting analysis of HeLa cells after S1P treatment for 5 min. Cytoplasmic and nuclear extracts were obtained and western blotting analysis conducted with the antibodies indicated (n = 3). E) Western blotting analysis of HeLa cell extracts after 5‐ and 10‐min treatment with S1P (10 µM). GAPDH and histone H3 (H3) antibodies were used as internal controls for cytoplasmic and nuclear extracts, respectively. All data are representative images of three independent experiments (n = 3). F) Pulldown assays using S1P immobilized on agarose beads or equivalent control beads. After extensive washing, bound proteins were dissolved in SDS sample buffer and separated by SDS‐PAGE and subjected to western blotting analysis (n = 3). C, Control; T, TCDF treated, respectively. Images are representative of three independent experiments.

Phosphorylation of SPHK2 by ERK leads to increased SPHK2 enzymatic activity and conversion of sphingosine to sphingosine‐1‐phosphate (S1P), a bioactive molecule indispensable for various cellular processes.^[^ ^34^ ^]^ We determined whether activation of the AHR promotes SPHK2 phosphorylation in the nucleus, and subsequent synthesis of nuclear S1P. Immunocytochemical analysis revealed p‐SPHK2 accumulation in the nucleus of HeLa cells after activation of AHR with TCDF (**Figure** **3**A; Figure S7, Supporting Information). Phosphorylation of SPHK2 occurred within 5 min after ligand activation (Figure 3B). In order to verify this result, we examined frozen liver tissue sections obtained from mice treated orally with TCDF. The TCDF treated mouse livers showed nuclear accumulation of AHR and p‐SPHK2 (Figure S8, Supporting Information) consistent with the cell culture results. Next, we analyzed the impact of S1P on AHR gene expression and protein levels and found that expression of *AHR* and *SPHK2* mRNAs were increased compared to controls (Figure 3C). To examine protein localization, cytoplasmic/nuclear extract fractionation and western blot analyses were performed. Consistent with changes in gene expression, S1P increased AHR and SPHK2 protein levels within 5 min particularly in nuclear extracts (Figure 3D). Prolonged treatment with S1P resulted in decreased AHR protein levels (Figure 3E), suggesting a transient mechanism by which p‐SPHK2/S1P can cause increased AHR retention in the nucleus. Furthermore, pulldown assays using S1P immobilized on agarose beads revealed a clear interaction between the AHR‐S1P complex in TCDF‐treated nuclear extracts (Figure 3F).

S1P signaling mainly acts through five G protein‐coupled S1P receptors (S1P1‐5) which are expressed ubiquitously.^[^ ^35^ ^]^ Considering the expression of S1P receptors under normal conditions, our results for AHR activation by S1P could be the consequence of S1P canonical signaling which is mediated through S1P receptors on the cell membrane. To address this possibility, we used FTY720 (fingolimod) which is a nonselective modulator of S1P receptors (S1P1, S1P3, S1P4, and S1P5).^[^ ^36^ ^]^ FTY720 induces the internalization and degradation of S1P1, and consequently inhibits S1P‐S1P receptor signaling. We first evaluated the cytotoxicity of FTY720 for HeLa cells and determined its optimal concentration as 5 µM (Figure S9A, Supporting Information). We confirmed that FTY720 (5 µM) treatment for 1 h caused clear S1P1 downregulation compared with control cells on their cell surfaces (Figure S9B, Supporting Information). Under the FTY720 treatment condition, the addition of S1P promoted AHR and SPHK2 expression especially in the nucleus compared to control implying that nuclear S1P signaling occurs quickly and independent of the S1P receptor pathway. Prolonged incubation of S1P following FTY720 treatment also resulted in downregulation of AHR expression in nucleus (Figure S9C, Supporting Information). Collectively, these results demonstrate that the synthesis of S1P by SPHK2 can rapidly regulate intracellular AHR levels in the nucleus.

### 2.5 Inhibition of *de novo* Ceramide Biosynthesis Decreases AHR Expression

**Figure 4:**
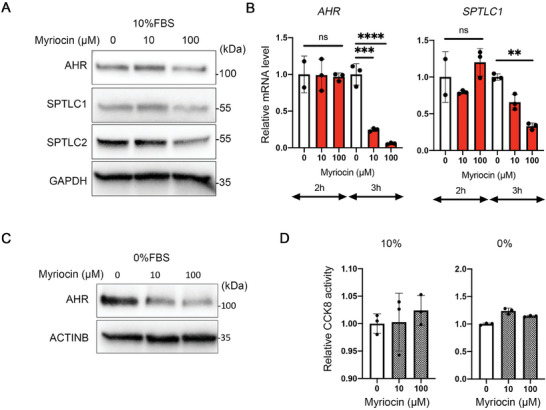
Inhibition of serine palmitoyltransferase with myriocin causes decreased AHR expression. A) Western blotting analysis of HeLa cells after myriocin (µM) treatment for 3 h. SPTLC1 and SPTLC2 antibodies were used for the conformation of myriocin effects for the inhibition under this culture condition (10%FBS/DMEM). B) qPCR analysis using *AHR‐ and SPTLC1‐*specific primers for 2 or 3 h after myriocin treatment. C) Western blotting analysis of HeLa cell whole lysates after myriocin (µM) treatment under serum free conditions for 1 h. D) CCK8 assay results after overnight culture in 10%FBS/DMEM or 2 h in 0%FBS/DMEM with or without myriocin. All data and images are representative from three independent replicates. ^****^ *p* < 0.0001, ^***^ *p* < 0.001, ^**^ *p* < 0.01.

Previously, we and others reported that AHR regulates expression of ceramide synthesis genes.^[^ ^19^, ^20^ ^]^ Furthermore, it was reported that *de novo* biosynthesis of sphingolipids is important for *CYP1A1* induction.^[^ ^37^ ^]^ We hypothesized that sphingolipid biosynthesis regulates AHR expression and in turn sphingolipid and ceramide *de novo* synthesis is regulated by AHR. To test this hypothesis, we examined expression of the AHR after inhibition of serine palmitoyltransferase with myriocin.^[^ ^38^ ^]^ Expression of both SPTLC proteins was reduced, confirming inhibition of serine palmitoyltransferase by myriocin and consistent with the inhibition of *de novo* ceramide biosynthesis. Inhibition of serine palmitoyltransferase with myriocin also caused a decrease in AHR protein expression 3 h after treatment compared to controls (**Figure** **4**A). Expression of *AHR* mRNA was also decreased as a result of inhibiting serine palmitoyltransferase (Figure 4B). *AHR* and *SPTLC1* mRNAs were decreased at 3 h consistent with the western blot results. Interestingly, the effects of inhibiting serine palmitoyltransferase with myriocin on AHR levels were more rapid if the cells were deprived of serum. A rapid decrease of AHR protein was found after 1 h in culture medium containing 0% FBS and myriocin (Figure 4C) with no apparent cytotoxicity (Figure 4D). Collectively, these results suggest that the *de novo* ceramide biosynthesis pathway is indispensable for the maintenance of AHR expression and signaling.

### 2.6 SPHK2/S1P Is Present on the *CYP1A1* Promoter Upon AHR Occupancy

**Figure 5:**
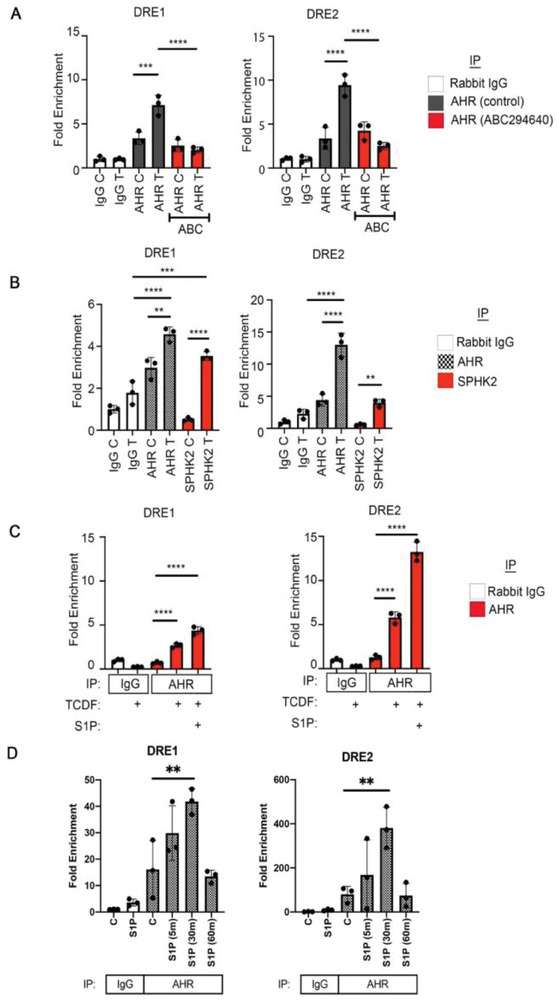
Characterization of SPHK2 and AHR occupancy on the *CYP1A1* promoter. A) ChIP‐qRT‐PCR analysis for the presence of the AHR in the proximity of the two DRE in the *CYP1A1* promoter in HeLa cells treated with or without 0.1 µM TCDF for 30 min or SPHK2 inhibitor ABC294640 (30 µM) pretreatment for 3 h followed by TCDF treatment for 30 min. Normal rabbit IgG was used for the negative control. C, control; T, TCDF; ABC, ABC294640. B) ChIP‐qPCR analysis for AHR and SPHK2 occupancy on the *CYP1A1* promoter in HeLa cells treated with or without TCDF (0.1 µM) for 5 min. C, control; T, TCDF. C) ChIP‐qPCR analysis for AHR occupancy proximal to each DRE in the *CYP1A1* promoter in HeLa cells with or without TCDF (0.1 µM) or S1P (1 µM) for 5 min. D) ChIP‐qPCR analysis for AHR occupancy proximal to each DRE in the *CYP1A1* promoter in HeLa cells with or without S1P (1 µM) for 5, 30, 60 min. All data are representative of independent three experiments. ^****^ *p* < 0.0001, ^***^ *p* < 0.001, ^**^ *p* < 0.01. ^*^ *p* < 0.05.

The downstream AHR target gene *CYP1A1* is upregulated when the AHR/ARNT/ligand complex is occupied on DREs in its promoter/enhancer region. Primers flanking the conserved DREs found in human and mouse *CYP1A1* promoters were designed (Figures S1 and S2 and Table S1, Supporting Information) and ChIP assays were performed using cells treated with ABC294640 to assess whether SPHK2 inhibition affects AHR recruitment to the *CYP1A1* promoter (**Figure** **5**A). These studies revealed that AHR occupancy was diminished by inhibiting SPHK2, suggesting that SPHK2 has a role in AHR binding to the DRE and target gene activation.

The presence of SPHK2 on the DREs was examined using an SPHK2 antibody and revealed that activating the AHR with TCDF increased the occupancy of SPHK2 on the *CYP1A1* DREs (Figure 5B). We also investigated whether S1P influenced the presence of AHR on the *CYP1A1* promoter. Cotreatment of S1P and TCDF rapidly increased AHR recruitment to the *CYP1A1* promoter (Figure 5C). Next, we investigated the effect of S1P on AHR recruitment to the *CYP1A1* DREs (Figure 5D). S1P at 1 µM increased AHR occupancy of the DREs in HeLa cells and Huh7 cells (Figure 5D; Figure S10A, Supporting Information), although no significant change in *CYP1A1* gene expression was observed (Figure S10B, Supporting Information). These results suggest a crucial and unique function for S1P production by SPHK2 in AHR occupancy on the promoter region containing DREs.

### 2.7 S1P Rescues *CYP1A1* Expression in CRISPR‐Cas9‐Mediated *SPHK2* Knockout Cells

**Figure 6:**
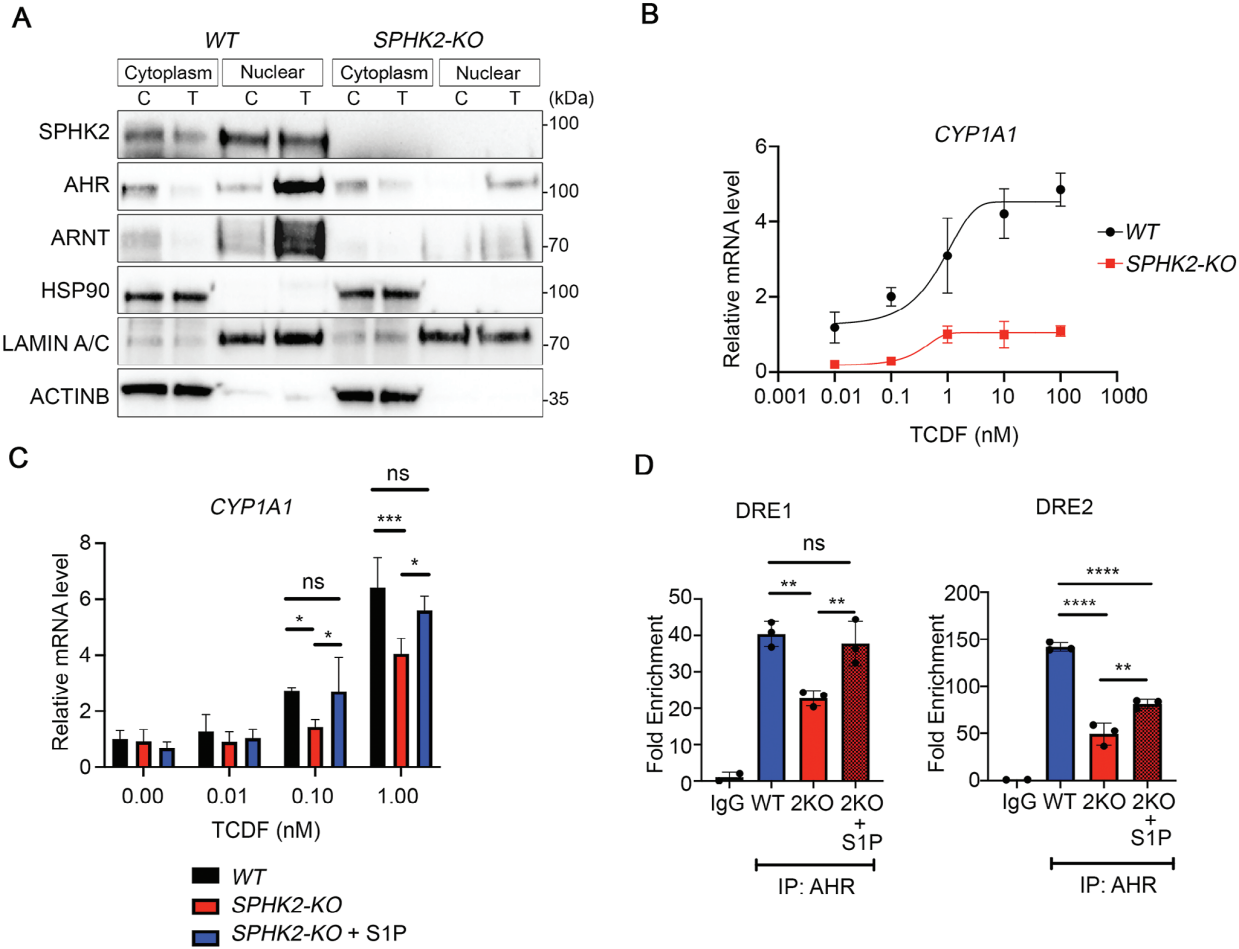
S1P rescues the downregulation of *CYP1A1* in CRSPR/Cas9‐mediated SPHK2‐KO cells. A) Western blotting analysis of HeLa CRSPR/Cas9‐treated cells after 30 min treatment with/without TCDF (0.1 µM), WT control or SPHK2‐KO HeLa cells were fractionated to isolate cytoplasmic and nuclear extracts and protein expression was assessed using antibodies as indicated. C, Control; T, TCDF treated, respectively. B) qPCR analysis of *CYP1A1* expression in WT control HeLa cells or SPHK2‐KO HeLa cells 2 h after dose‐dependent TCDF exposure. C) qPCR analysis for *CYP1A1* expression in WT control HeLa cells, SPHK2‐KO HeLa cells, and SPHK2‐KO+S1P HeLa cells. Cells treated with TCDF (0.1 and 1 nM). D) ChIP‐qPCR analysis for the presence of the AHR in the two DRE in the *CYP1A1* promoter in WT control or SPHK2‐KO HeLa cells treated with or without 1 µM S1P for 5 min. Normal rabbit IgG was used for the negative control. C, control; T, TCDF; ABC, ABC294640; 2KO, SPHK2‐KO. All data are representative of three independent experiments. ^****^ *p* < 0.0001, ^***^ *p* < 0.001, ^**^ *p* < 0.01. ^*^ *p* < 0.05.

We next used CRISPR/Cas9‐mediated *SPHK2* knockout (KO) HeLa cells^[^ ^32^ ^]^ to examine the role of S1P on AHR signaling. We verified knockdown efficiency of SPHK2 by western blot analysis and confirmed that CRISPR/Cas9‐mediated SPHK2 knockdown significantly diminished SPHK2 protein expression (**Figure** **6**A). Moreover, AHR and ARNT levels, but not HSP90, were significantly decreased by knocking out the *SPHK2* gene. We also analyzed the dose‐dependent change in *CYP1A1* expression after activation of the AHR with TCDF (Figure 6B). In wild‐type HeLa cells, activating the AHR with TCDF dose‐dependently induced *CYP1A1* mRNA from 0.01 to 10 µM, while this effect was diminished in *SPHK2*‐KO HeLa cells (Figure 6B).

Rescue experiments using S1P supplementation were also examined. Low doses of S1P increased *CYP1A1* mRNA levels in *SPHK2*‐KO cells compared to controls (Figure 6C). Furthermore, ChIP analysis of the two DRE regions of the *CYP1A1* promoter demonstrated that S1P (1 µM) treatment partially restored AHR recruitment to the DREs in *SPHK2*‐KO HeLa cells (Figure 6D). These data collectively demonstrate that SPHK2/S1P has a crucial role in the regulation of *CYP1A1* expression by enhancing AHR promoter occupancy.

### 2.8 SPHK2 Regulates AHR Translocation into the Nucleus Through the LXXLL Consensus Motifs

**Figure 7:**
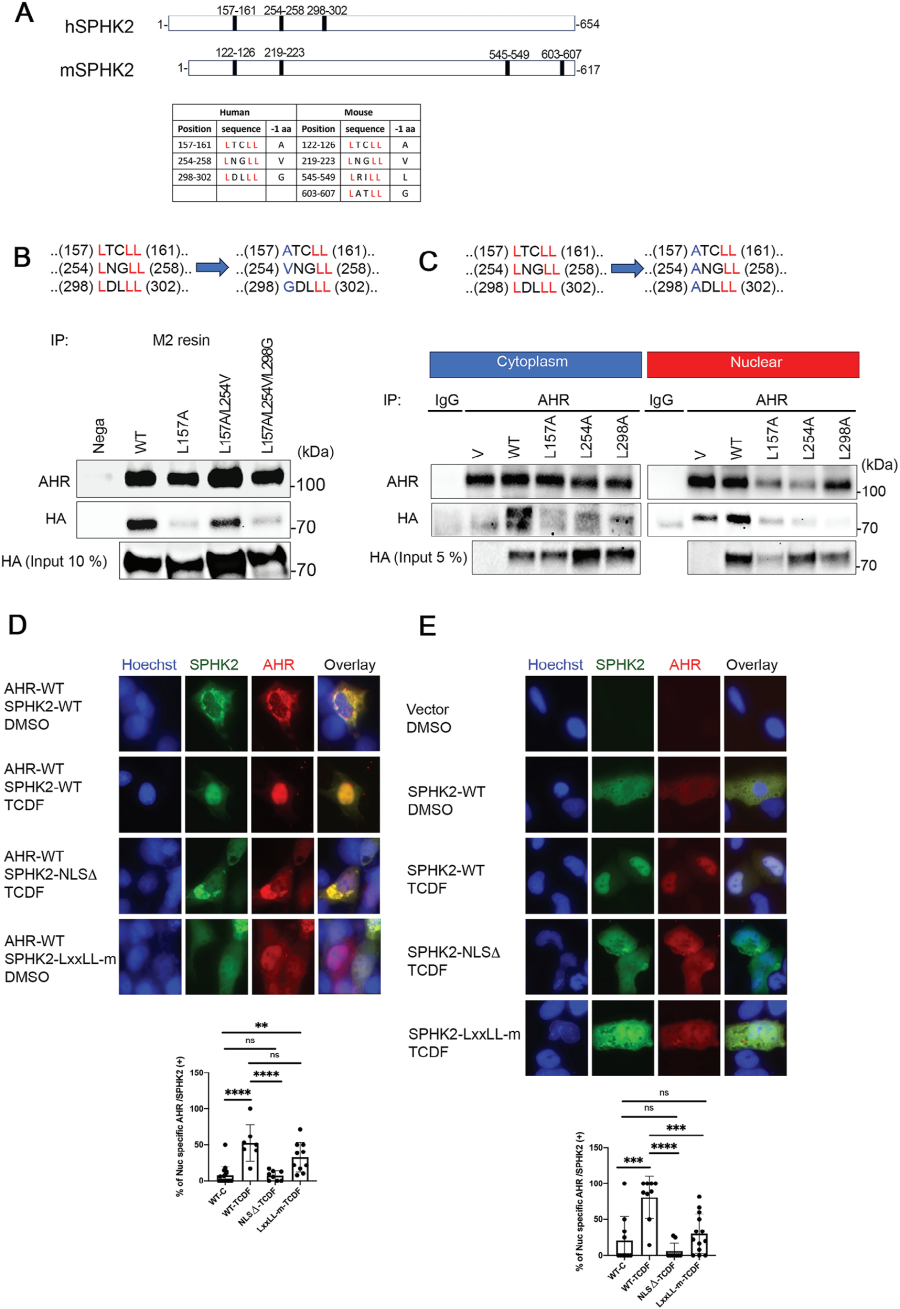
The LXXLL motif facilitates the binding of SPHK2 to AHR in the nucleus. A) Alignment of LXXLL motif sequences present in human and mouse SPHK2. B) (Top)Scheme of site‐directed mutagenesis of LXXLL motif. Three of the human SPHK2 LXXLLs motif are indicated. Numbering indicates the position of the amino acid substitution. (Bottom) Co‐IP image of nuclear extracts of COS‐1 cells that were transfected with hAHR‐FLAG and hSPHK2‐HA WT or LXXLL single, double, and triple mutation plasmids for 48 h followed by TCDF treatment for 30 min. C) (Top)Scheme of site‐directed mutagenesis of LXXLL motif. (Bottom) Co‐IP image of cytoplasm and nuclear extracts of COS‐1 cells after transfection with both hAHR‐FLAG and hSPHK2‐HA WT or SPHK2‐LXXLL single mutation plasmids for 48 h followed by TCDF treatment for 30 min. V, control vector. D) Immunofluorescence images of COS‐1 cells co‐transfected with AHR‐FLAG and either SPHK2‐WT, SPHK2‐NLS deletion (NLSΔ), or SPHK2‐triple LXXLL mutation plasmids (LXXLL‐m). (Bottom) Quantitative analysis of the percentage of AHR+ nuclear per SPHK2 + cells in randomly selected independent fields. n > 200 cells (fields n > 5). E) Immunofluorescence images of SPHK2‐KO HeLa cells transfected with control vector (Vector), SPHK2‐WT, SPHK2‐NLS deletion (NLSΔ), or SPHK2‐triple LXXLL mutation plasmids (LXXLL‐m). (Bottom) Quantitative analysis of percentage of AHR+ nuclear staining per SPHK2 + cells in randomly selected independent fields. n > 200 (fields n > 13). Bar = 20 um.

Next, we assessed if LXXLL motifs in the SPHK2 structure were dispensable for AHR binding and if they influenced nuclear translocation. Human SPHK2 has three LXXLL motifs in the N terminal region and mouse SPHK2 has two in the N terminus and two in C terminus, for a total four LXXLL motifs (**Figure** **7**A). Among them, the first two N terminal LXXLL motifs (human 157–161 and mouse 122–126 and human 254–258 and mouse 219–223) are highly conserved including a hydrophobic residue at −1 position.

We generated a plasmid using site‐directed mutagenesis with a single or multiple mutations in the two conserved LXXLL sequences (Figure 7B,C), co‐transfected into COS‐1 cells with human AHR plasmid (pcDNA3‐hAHR‐FLAG) and performed co‐IP after AHR activation with TCDF treatment to activate the AHR (Figure 7B). IP‐western blotting results show that the single and triple mutant forms of LXXLL motifs significantly reduced the interaction with the AHR in the nucleus. We also replaced Leu254 and Leu298 with Ala and performed the same experiments confirming that all Ala mutants had reduced AHR interaction both in the cytoplasm and nucleus (Figure 7C).

We also examined the influence of the nuclear localization sequence (NLS) mutant SPHK2 on the localization of AHR in COS‐1 cells. The NLS in SPHK2 was deleted^[^ ^30^ ^]^ and co‐transfected with AHR‐FLAG (Figure 7D). In control experiments, co‐transfected AHR and wild‐type SPHK2, the two proteins exhibited increased colocalization in the nucleus after activation of the AHR with TCDF (top and second row panels). However, AHR translocation into the nucleus was largely compromised by co‐transfection with the NLS‐deleted form of SPHK2 (third row). In addition, the triple LXXLL mutated (L157A/L254V/L298G) form of SPHK2 also caused reduced translocation of AHR to the nucleus (bottom row).

We also transfected a series of SPHK2 plasmids into CRISPR‐SPHK2‐KO of HeLa cells (Figure 7E). Of note, endogenous AHR immuno‐staining was very low in control vector transfected *SPHK2*‐KO cells, supporting results described above (top row). SPHK2‐WT transfected cells exhibited enhanced immunohistochemical staining of SPHK2 and AHR (second row) and AHR translocation into the nucleus after TCDF activation (third row). Similar to the results observed in COS‐1 cells, SPHK2‐NLS deletion and triple LXXLL mutation of SPHK2 reduced the translocation of AHR into the nucleus (4th and 5th row).

Collectively these results demonstrate that SPHK2 has a crucial function in the regulation of AHR translocation and subsequent downstream AHR target gene activation through LXXLL consensus sequences.

### 2.9 Activated AHR Regulates SPHK2 Translocation into the Nucleus Highlighting the Importance of Reciprocal Interactions Between AHR and SPHK2

Although our data suggest that AHR activation upregulates p‐SPHK2 levels in the nucleus (Figure S8, Supporting Information), we wanted to know if phosphorylation of SPHK2 still occurs under AHR knockdown conditions. First, we assessed SPHK2 translocation into the nucleus using an AHR‐NLS deletion plasmid which compromises AHR translocation into the nucleus from the cytoplasm (Figure S11, Supporting Information). Interestingly, without AHR translocation, SPHK2 was restricted in the cytoplasm (Figure S11A, Supporting Information, arrows).

Furthermore, we used *Ahr*‐null mice to verify the role of AHR for SPHK2 phosphorylation and nuclear translocation. In wildtype mice, dietary TCDF administration enhanced p‐SPHK2 expression in the nucleus (Figure S12, Supporting Information, white arrowheads). In *Ahr‐*null mouse liver, p‐SPHK2 expression could be detected mainly in the cytoplasm and moderately in nucleus (Figure S12G, Supporting Information). However, p‐SPHK2 in *Ahr*‐null mouse livers following TCDF treatment remained in the cytoplasm (Figure S12H, Supporting Information, red arrowheads). These results support that AHR activation can also regulate SPHK2 translocation, and that reciprocal interactions between AHR and SPHK2 is needed for AHR downstream target genes activation.

## 3 Discussion

**Figure 8:**
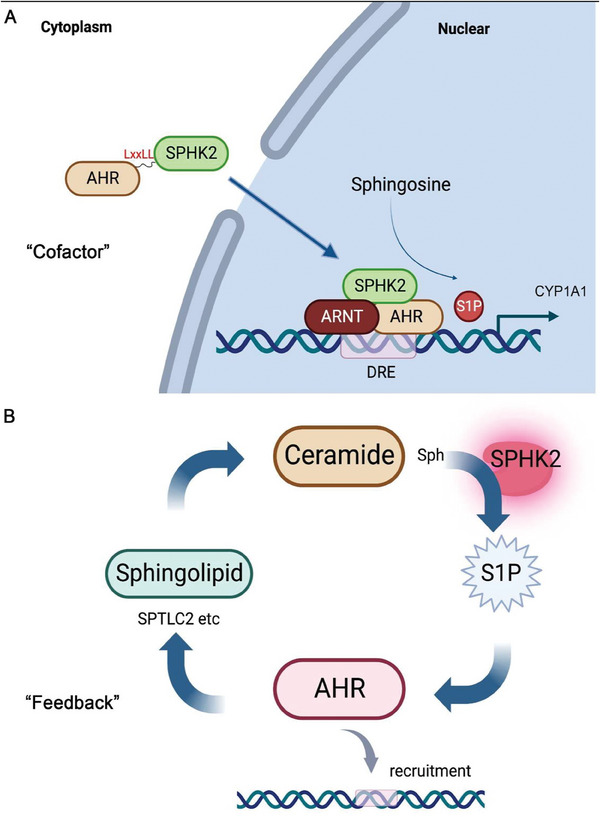
Schematic illustration of the dual function of SPHK2. A) SPHK2 functions as a cofactor for the AHR/ARNT heterodimer on the DRE‐containing promoter region of the *CYP1A1* gene. B) SPHK2, S1P, and AHR establish a positive feedback mechanism for ceramide de novo biosynthesis metabolism. S1P also enhances AHR recruitment to DREs.

This study revealed that SPHK2 interacts with the AHR in the nucleus and is a critical cofactor that modulates AHR activity. SPHK2 silencing decreased AHR levels and signaling while overexpression of SPHK2 increased AHR expression. Moreover, SPHK2 is enriched on the *CYP1A1* promoter region containing DREs indicating an unexpected role for SPHK2 as a co‐activator of transcription in AHR signaling. In addition, S1P rapidly increases not only AHR expression at the mRNA and protein levels but also recruitment and occupancy of the AHR on target gene DREs. These unexpected dual roles for SPHK2 are summarized in **Figure** **8**.

Previous reports suggest that phorbol 12‐myristate 13‐acetate (PMA) increases S1P in the nucleus within 5 min and rapidly enhances the colocalization of SPHK2 and histone deactetylases (HDAC), while prolonged treatment induces nuclear export of SPHK2, ensuring transient inhibition of HDAC by SPHK2.^[^ ^34^ ^]^ In the present study, we also demonstrated that transient stimulation of S1P increases AHR and *CYP1A1* gene expression, while prolonged exposure to S1P decreases their expression. This may be because S1P is incorporated into the sphingolipid metabolic pathway and is also converted to other lipid metabolites that could influence AHR expression and signaling.

SPHK2 generates S1P that functions in a variety of cellular events.^[^ ^34^, ^39^ ^]^ Our results strongly support dual roles for SPHK2 as an enzyme that modulates ceramide levels and regulates AHR signaling. Importantly, a role for sphingolipids in gene regulation was reported in various biological events. For example, ceramide synthase Schlank, which is an orthologue of mammalian ceramide synthase 2 (CERS2) was reported to participate in transcriptional regulation at the chromatin level.^[^ ^21^ ^]^ Interestingly, this mechanism is conserved in mammalian CERS2, emphasizing the importance of CERS proteins in gene regulation.^[^ ^40^ ^]^ Furthermore, sphingosine, which is metabolized by SPHK1/2 enzymes to generate S1P, was shown to bind to steroid factor‐1 in the nucleus and regulate the expression of target genes.^[^ ^41^ ^]^ These findings suggest the conserved multiple functions of sphingolipid enzymes in transcriptional regulation, which is an unexplored topic.

Recent reports suggest that SPHK2 is present on the promoters of hypoxia‐inducible factor (HIF1) target genes and that nuclear S1P regulates not only the expression of downstream target genes but also the expression of HIF1α via a positive feedback mechanism.^[^ ^42^ ^]^ Our findings for AHR are similar to these findings for HIF1α. Considering that ARNT forms a heterodimer with both AHR and HIF1α, and our results suggest direct interactions among these three proteins, it is likely that the SPHK2/S1P complex is recruited into both pathways in a cell context‐dependent manner. Further experiments regarding the crosstalk between these two pathways will be key to understanding this signaling.

Bioactive sphingolipids such as ceramides and S1P are tightly regulated. Accumulating evidence suggests that an imbalance of sphingolipids may play a role in neurodegenerative disorders such as Alzheimer's, Parkinson's, Huntington's, and Amyotrophic Lateral Sclerosis (ALS).^[^ ^43^ ^]^ In the current study, we demonstrate that SPHK2 not only works as a cofactor of AHR/ARNT for downstream target gene activation, but also enhances AHR‐mediated target gene expression. These results suggest a critical role for SPHK2 and S1P in AHR transcriptional activity, which potentially provides a link between ceramides/S1P and commonly regulated critical cellular functions, such as the programming of keratinocyte or intestinal epithelial cell differentiation.^[^ ^17^, ^18^ ^]^

Using our survey of LXXLL motifs in sphingolipid and ceram18ide proteins and co‐IP experiments, we showed that SPHK2 has multiple LXXLL motifs in its amino acid sequence and has a pivotal role in regulating AHR nuclear import upon interaction with ligand‐activated AHR. Interestingly, a previous report suggested that CERS6, a rate limiting sphingolipid for the *de novo* ceramide biosynthesis enzyme, is expressed predominantly in the perinuclear region and regulates the nuclear translocation of active caspase‐3 involved in the control of cell apoptosis.^[^ ^44^ ^]^ It is also known that cancer cells tightly regulate ceramide signaling.^[^ ^45^ ^]^ Notably, as a mechanism for aberrant growth, cancer cells upregulate enzymes that metabolize ceramides because these lipids can cause cancer cell programmed cell death. Based on multiple reports of sphingolipid ceramide involvement in the regulation of cancer cells, it is likely that other sphingolipids that are not yet reported also modulate cancer cell survival through the LXXLL consensus sequence in the nucleus.

Furthermore, we identified SGPL1 (sphingosine‐1‐phosphate lyase 1) as another candidate with potential interaction with nuclear receptors/transcription factors (Table S3, Supporting Information). SGPL1 cleaves S1P to yield long‐chain aldehyde and ethanolamine phosphate.^[^ ^46^ ^]^ The precise regulation of nuclear S1P production via AHR interaction with SPHK2/SGPL1 may affect AHR and downstream target gene activation.

This work sheds light on the crosstalk between sphingolipid proteins, sphingolipids, and transcription factors at the gene regulation level, which has not been studied in detail. It also suggests that this mechanism could be broadly conserved, and various sphingolipid proteins could be participating in the fine‐tuning of gene regulation at the chromatin level in the nucleus and likely regulating important biological activities, such as cell differentiation.

## 4 Experimental Section

### Reagents

Main reagents used in this manuscript were as follows; 2,3,7,8‐Tetrachlorodibenzofuran (TCDF, #EF‐903‐C, Cambridge Isotope Laboratories), myriocin (from Mycelia sterilia, #M1177, SIGMA), S1P bioactive lipid (#73 914, SIGMA), SPHK2 inhibitor ABC294640 (C_23_H_25_ClN_2_O, #B‐0025, Echelon Biosciences Inc.). Indolo[3,2‐b] carbazole (ICZ, #02 4785, Matrix Scientific). FTY720 (#SML0700, SIGMA).

### Expression Vectors

Human AHR plasmid (pcDNA3‐hAHR‐FLAG) was reported previously.^[^ ^47^ ^]^ Human NLS deleted AHR plasmid was reported previously.^[^ ^27^ ^]^ HA tagged human SPHK2 vectors (p‐PM‐C‐HA) were purchased from Applied Biological Material Inc. 4 unique 29mer shRNA for human SPHK2 shRNA plasmids (pGFP‐V‐R) were purchased from Origene. Specific sequences used for shRNA are shown in Table S2 (Supporting Information).

### Antibodies

Primary antibodies used were as follows: AHR: AHR Rabbit mAb (D5S6H, Cell Signaling technology), Goat anti‐AHR antibody (#NB100‐128, Novus Biologicals), Mouse monoclonal Anti‐AHR #14‐9854‐82, Invitrogen), ARNT: HIF‐1b/ARNT Rabbit mAb (D28F3, Cell Signaling Technology), SPHK2: SPHK2 Rabbit mAb (D2V3G, Cell Signaling Technology), Phospho‐SPHK2: Anti‐Phospho‐SphK2 (T614) Antibody (#A01382T614, Bosterbio), SPTLC1: anti‐SPTLC1 antibody (ab176706, abcam), SPTLC2: anti‐Serine Palmitoyltransferase antibody (ab236900, abcam), SPHK1: SPHK1 (D1H1L) Rabbit mAb  (#12 071, Cell Signaling Technology), S1P1: S1P1 Polyclonal Antibody (#PA1‐1040, Thermo Scientific), Actinb: β‐Actin (13E5) Rabbit mAb (#4970, Cell signaling Technology), GAPDH: GAPDH (D16H11) XP Rabbit mAb (#5174, Cell Signaling Technology), Histon H3: Histone H3 (D1H2) XP Rabbit mAb (#4499, Cell Signaling Technology), HA: HA‐Tag Rabbit mAb (C29F4, Cell Signaling Technology).

Secondary antibodies used were as follows; Mouse Anti‐rabbit IgG (Conformation Specific) mAb (HRP Conjugate) (L27A9, Cell Signaling Technology), Rabbit Anti‐Mouse IgG (Light Chain Specific) mAb (HRP Conjugate) (D3V2A, Cell Signaling Technology). Goat anti‐Rabbit IgG (H+L) Cross‐Adsorbed Secondary Antibody, Alexa Fluor 488 (#A11008, Invitrogen), Donkey anti‐Goat IgG (H+L) Highly Cross‐Adsorbed Secondary Antibody, Alexa Fluor Plus 488 (#A11055, Invitrogen), Donkey anti‐Goat IgG (H+L) Highly Cross‐Adsorbed Secondary Antibody, Alexa Fluor Plus 594 (#A32758, Invitrogen), Donkey polyclonal Anti‐Rabbit IgG (H+L) Highly Cross‐Adsorbed Secondary Antibody, Alexa Fluor 594 (#A21207, Invitrogen),  Donkey anti‐Mouse IgG (H+L) Highly Cross‐Adsorbed Secondary Antibody, Alexa Fluor 488 (#A21202, Invitrogen), Hoechst33342 (#62 249, Thermo Scientific).

### Mice and Diet

TCDF treatment in mice was performed according to a previous report.^[^ ^48^ ^]^ Briefly, female C57BL/6J wild type mice were fed dough diets (#S3472, Bio‐Serv) containing TCDF (24 µg k^−1^g) or control. After 24 h, mice were sacrificed and liver tissue harvested. The left liver lobe was removed and embedded in OCT compound and stored at −80 °C until use. Frozen sections (10 µm thickness) was made using Leica CM1950 Cryostat. *Ahr*‐null mice were provided by Dr. Christopher Bradfield, bred in house, and treated as above. The experiments were performed using protocols approved by the Pennsylvania State University (PROTO202001416) Institutional Animal Care and Use Committee.

### Cell Culture and TCDF Treatment

HeLa cells (human cervical cancer cells), Huh7 (human hepatoma cells) or COS‐1 cells (monkey kidney cells) were grown in 10 mm or 15 mm plastic dishes (Falcon) containing DMEM (Gibco DMEM, high glucose #11965‐092, Thermo Fisher Scientific) supplemented with 10% Fetal bovine serum (FBS, heat‐inactivated at 56 °C, #S11150H, R&D Systems) and 1% penicillin/streptomycin (#15140122, Gibco) at 37 °C 5% CO_2_ in a humidified chamber. After reaching 90–100% confluency, 2,3,7,8‐tetrachlorodibenzofuran (TCDF) dissolved in DMSO was added into the culture medium at a final concentration of 0.1 µM and incubated. An equal amount of DMSO was added into the medium as control.

### CRSPR/Cas9 Mediated SPHK2 Knockout Cells

CRSPR/Cas9 mediated human *SPHK2* knockout (KO) cells were prepared as previously described.^[^ ^32^ ^]^ Complete loss of SPHK2 protein was verified by western immunoblot analysis. Cells were cultured in 10% FBS/GlutaMAX‐DMEM (#10569010, Thermo Fisher Scientific) and 1% Penn/Strep as described. Before each analysis, all antibiotics were removed from the media. For S1P treatment, SPHK2‐KO HeLa cells were cultured in 12 well plates (1.0 × 10^5^ per well) for 24 h. After treatment with TCDF (0.1 and 1 nM) for 30 min, 10 nM S1P was added to each well and further incubated for 90 min.

### Plasmid Transient Transfection

HeLa cells were grown in 10% FBS/DMEM in 10 cm dish until 70–80% confluent. Transient transfection of 4–8 µg plasmid was performed using Lipofectamine 3000 (Thermo Fisher Scientific) according to the manufacturer's protocol. After 48 h following transfection, the cells were harvested with Trypsin‐EDTA, washed with PBS, and pelleted at 100 g for 5 min for cytosol/nuclear fractionation.

### Generation of LXXLL Mutation Plasmid (Site‐Directed Mutagenesis Assay)

Site directed mutagenesis of human SPHK2 was performed using Q5 Site‐directed mutagenesis kit (#E0554S, New England Biolabs) and pPM‐hSPHK2‐HA as a template following manufacturer's instructions. For LXXLL consensus motifs mutation, first leucine residue of three LXXLL motifs in human SPHK2 (Table S2, Supporting Information) were replaced with alanine (A), valine (V) or glycine (G), respectively, or all replaced with A. The primers used are as follows; (L157A) 5′‐GGCCACTGCCGCGACCTGTCTGC‐3′ and 5′‐CAGCGCTGGGCCTCG‐3′. (L254V) 5′‐CCATGAGGTGGTGAACGGGCTCC‐3′ and 5′‐AGCAGCCCGTCTCCC‐3′. (L298G) 5′‐AGCCCTGGGCGGCGACCTGTTGC‐3′ and 5′‐GGCTCAAATCCCCCGTG‐3′. (L254A) 5′‐CCATGAGGTGGCGAACGGGCTCC‐3′ and 5′‐AGCAGCCCGTCTCCC‐3′. (L298A) 5′‐AGCCCTGGGCGCGGACCTGTTGC‐3′ and 5′‐GGCTCAAATCCCCCG‐3′. NLS‐deletion of hSPHK2 was performed using mutagenic synthetic oligonucleotides (5′‐GCC ACT CGC ACC TTC‐3′) and its reverse complement which deleting the putative NLS region consensus sequence (residues 86–94, RGRRGARRR^[^ ^30^ ^]^). Each mutant was confirmed by DNA sequencing.

### Cytoplasmicl/Nuclear Extract Fractionation of Cultured Cells

Cytoplasmic and nuclear extract fractionation of cultured cells were performed according to previous reports.^[^ ^49^ ^]^ Briefly, following administration of TCDF, ICZ or carrier solvent, cells were washed with cold PBS and harvested with Trypsin‐EDTA, wash twice with PBS, and homogenized in 1 mL of MENG buffer (25 mM MOPS, 2 mM EDTA, 0.02% NaN3, 10% glycerol, 20 mM sodium molybdate) containing proteinase inhibitors (complete, Mini Protease Inhibitor Cocktail, #11836170001, SIGMA) and 1 mM phenylmethylsulphonyl fluoride (PMSF) using a 7 ml Dura‐Grind Dounce stainless steel homogenizer (Wheaton). Nuclei were isolated by centrifugation of the cell lysates at 1000 g for 20 min. The supernatant was moved to 12 mL round bottom ultracentrifuge tubes and centrifuged at 100 000 g for 1 h to obtain the cytosolic fraction. Nuclei were resuspended and washed three times with MENG buffer and the nuclei resuspended in MENG containing 500 mM NaCl, incubated on ice for 1 h with gentle vortex every 10 min, and then centrifugation at 100 000 g for 30 min to obtain nuclear extract. Total protein in cytosolic and nuclear fractions was determined with the Pierce BCA Protein Assay kit (#23225, Thermo Fisher Scientific).

### Immunoprecipitation (IP) Assay

Protein G Magnetic beads (30 µl) (#S1430S, BioLabs) washed twice with TBS (50 mM Tris, 150 mM NaCl) were incubated with 1 µg of primary antibodies diluted 1:100. The mixture was incubated for 30 min at RT by gentle shaking. Each IP lysate was preincubated with Protein G beads for 60 min at 4 °C to remove background interactions. A total of 600–800 µg of protein was incubated with 30 µL Protein G and antibody mixture for 90 min at 4 °C with gentle rocking. At the end of incubation, samples were centrifuged and in the presence of a magnet, the supernatant was removed to retain the beads. The beads were washed with TBS three times for 10 min with shaking to remove non‐specific binding. Protein‐bound beads were dissolved in SDS sample buffer (5X, 300 mM Tris‐HCl, 1% 2‐mercaptoethanol, 40% glycerol, 5% SDS, 0.05% bromophenol blue) and used for western blotting analysis.

For site‐directed mutagenesis assay, both human AHR plasmid (pcDNA‐hAHR‐FLAG) and pPM‐hSPHK2‐HA or each mutant (L157A, L254V, L298G, L175A/L254V, L254V/L298G, L254A, L298A and L157A/L254V/L298G) were co‐transfected into COS‐1 cells using Lipofectamine 3000. After 48–72 h later, cells were treated with TCDF for 30 min, then cytoplasmic/nuclear extract fractionation was performed. After determination of protein concentrations, equal amounts of protein were incubated with Anti‐FLAG M2 magnetic beads (M8823, Sigma) for 2 h at 4 °C. Western blotting samples were prepared as described above.

### S1P Pulldown Assay

S1P‐immobilized agarose beads (#S‐2000) and the equivalent control beads (#P‐B000) were purchased from Echelon (Echelon Biosciences). The pulldown assay was performed according to the manufacturer's protocol and 20 µl of beads were incubated with 100 mg of total protein each for 3 h at 4 °C. The beads were boiled in SDS sample buffer for 5 min at 95 °C and the supernatants separated by SDS‐PAGE electrophoresis.

### Western Blotting

Samples were dissolved in SDS sample buffer and boiled for 5 min at 95 °C and then loaded onto a precast gel (4–20% Criterion TGX Precast Midi Protein Gel, #5671094 BioRad) followed by electrophoresis at 150 V for 90 min. Protein membrane transfer was performed using the Trans‐Blot Turbo Transfer System Transfer Pack (#1704157EDU, BioRad). After blocking with 5% non‐fat dry milk (#sc‐2325, Santacruz)/TBST (TBS‐0.1%Tween 20) for 60 min at RT, blots were incubated with primary antibodies in 5% non‐fat dry milk/TBST overnight at 4 °C with gentle shaking. Blots were washed with TBST several times and incubated with the appropriate secondary antibody in 5% non‐fat dry milk/TBST for 2 h at RT with gentle agitation. SuperSignal West Dura (#34075, Thermo Fisher Scientific) using ChemiDocTM XRS+ (BioRad) was used to image the blots.

### SPHK2 Inhibitor Assay

SPHK2 inhibitor (ABC294640) was dissolved in DMSO at 5 mg/ml and stored at −20 °C until use. For qRT‐PCR analysis, HeLa cells were cultured in 6 well plastic dishes (3 × 10^5^ cells per well) for 24 h and pretreated with ABC294640 or equivalent volume of DMSO as a control for 2 h, then 0.1 µM TCDF or DMSO was added for an additional 2 h. Cells were harvested using TRizol Reagent (#15596026, Thermo Fisher Scientific). For cytoplasmic/nuclear extract fractionation followed by western blot analysis, the cells were grown in 150 mm cell culture dishes (#353025, Falcon) until confluent. Cytoplasm and nuclear extracts were isolated as described above.

### CCK8 Cell Viability Assay

Cell viability assays were performed using Cell Counting Kit‐8 (CCK8) assay (#CK04‐11, Dojindo) according to the manufacturer's instructions. Briefly 5 × 10^3^ cells per well were cultured in a 96 well culture plate (Falcon) maintained in DMEM under serum free or 10% FBS. Cells were treated with various concentrations of ABC294640 and reacted with the CCK8 reagent for 60 min at 37 °C. The absorbance values were measured at 450 nm wavelength using a microplate reader (SYNERGY HTX, BioTek).

### Immunofluorescent Analysis

HeLa cells were cultured on chambered coverglass (#155383, Thermo Fisher Scientific) in 10% FBS/DMEM. After fixation with 10% formalin for 10 min at RT, cells were permeabilized with 100% MeOH at −20 °C for 10 min. Cells were blocked with 5% BSA, 5% donkey serum/PBS for 1 h at RT, incubated with primary antibody as described within each figure for 16 h at 4 °C. Alexa fluor 488/594 conjugated antibodies were used as secondary antibodies and nuclei were counterstained with Hoechst 33342. After extensive washing, samples were analyzed with a LSM880 confocal laser scanning microscope at the Penn State microscopy core facility.

### Quantitative PCR (qRT‐PCR) Assay

Total RNA of cultured cells was collected using TRIzol Reagent (Invitrogen). RNA was reverse transcribed with qScript cDNA Supermix (#95048, Quantabio). qPCR was performed using PowerUp SYBR Green master mix (Applied Biosystems). The primer sequences used for this study are as follows. *hSPHK2*: Forward‐GAGCCTGAGTGAGTGGGATG, Reverse‐CAGTCAGGGCGATCTAGGAG, *hAHR*: Forward‐ATTGTGCCGAGTCCCATATC, Reverse‐TGCATTAGACTGGACCCAAG, *hCYP1A1*: Forward‐GGTCAAGGAGCACTACAAAACC, Reverse‐TGGACATTGGCGTTCTCAT, *hSPTLC1*: Forward‐CTCAGGCACGGTACTTGGAC, Reverse‐GATGCAGCCCTCTGTAGCTC, *hGAPDH*: Forward‐TGTGGTCATGAGTCCTTCCACGA, Reverse‐AGCCTCAAGATCATCAGCAATGCC, all gene expression levels were normalized to that of GAPDH.

### Chromatin Immunoprecipitation (ChIP) Assay

Chromatin immunoprecipitation assays (ChIP) were performed using a SimpleChIP Enzymatic Chromatin IP kit (Magnetic Beads) (#9003, Cell Signaling Technology) according to the manufacturer's instructions. Briefly, HeLa cells treated with/without TCDF or ABC294640 were crosslinked with 1% formaldehyde solution (#0493, AMRESCO) for 10 min at RT. Antibodies against AHR or SPHK2 were incubated with extracted chromatin for 16 h with gentle rotating at 4 °C. After the elution of chromatin by gentle vortex (1200 rpm) for 30 m at 65 °C, samples were reverse cross‐linked by adding 5 M NaCl and Proteinase K for 2 h at 65 °C and purified with Spin column supplied in the kit. qPCR was used to quantitatively assess the promoter region of the *CYP1A1* gene found near the DREs. Primer design of DRE rich region of the *CYP1A1* promoter was conducted as follows: The mouse AHR ChIPseq dataset registered with NCBI GEO (GSE97634) was analyzed using the Integrated Genome Browser (https://www.bioviz.org/) to confirm the AHR binding peak of the *Cyp1a1* promoter (Figure S1, Supporting Information). The AHR binding sequences contained in the obtained peaks were analyzed using the JASPAR database (https://jaspar.genereg.net/) to identify six putative AHR binding sequences (Seq‐1, −2, −3, −4, −5 and −6) (Table S1, Supporting Information). These sequences were analyzed using the UCSC database (https://genome.ucsc.edu/) and three AHR/dioxin‐responsive elements (DRE‐1, −2 and −3) were identified that are conserved between human and mouse (Figure S2, Supporting Information). ChIP‐qPCR primers for the human *CYP1A1* promoter were designed to target two of these three DREs using Primer3Plus software (http://www.bioinformatics.nl/cgi‐bin/primer3plus/primer3plus.cgi). Primer sequences used for ChIP‐qPCR were shown in Table S2 (Supporting Information) as a key resource table.

### LXXLL Motif Analysis

Human and mouse sphingolipids, ceramide and related protein amino acid sequences were analyzed for Leu‐Xaa‐Xaa‐Leu‐Leu (LXXLL: Position +1–+5, L is leucine, x is any amino acid) helical motifs within the sequence. The amino acid at position −1 was annotated as a hydrophobic residue or basic amino acid.^[^ ^22^, ^23^ ^]^

### Statistical Analysis

Statistical analysis was performed using an unpaired two‐tailed student's *t*‐test for the two samples comparison, using Graphpad Prism 7.0. For all experiments, the data set from triplicate samples were calculated and expressed as mean ± standard deviation (SD). For multiple samples comparison, one‐way ANOVA test was utilized. The experiments were repeated three times and representative data were used *p* < 0.05 was considered statistically significant.

## Conflict of Interest

The authors declare no conflict of interest.

## Supporting information

Supporting Information

## Data Availability

The data that support the findings of this study are available from the corresponding author upon reasonable request.
