# Supplementary material for: Sphingosine Kinase 2 Regulates Aryl Hydrocarbon Receptor Nuclear Translocation and Target Gene Activation
Source: Adv Sci (Weinh). 2024 Aug 29;11(40):2400794. doi: 10.1002/advs.202400794 (PMC11516111; doi:10.1002/advs.202400794)

**Supplemental Information**

**
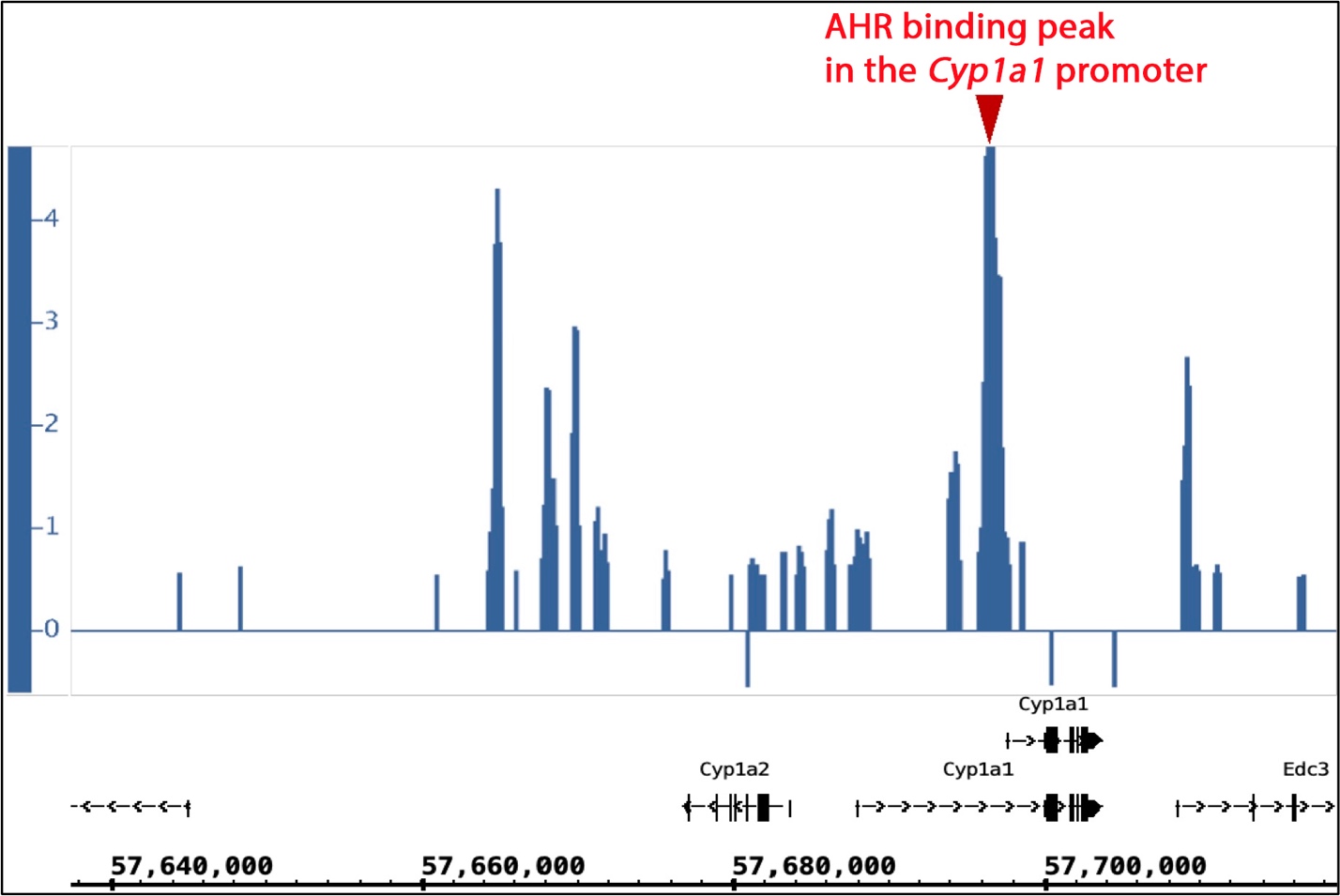
**

**Supplemental Fig. S1.**

Chromatin immunoprecipitation. AHR ChIP-seq reads peaks in the *Cyp1a1* gene from livers of mice gavaged with TCDD for 2 h. AHR ChIP-seq datasets were provided by NCBI GEO (GSE97634).


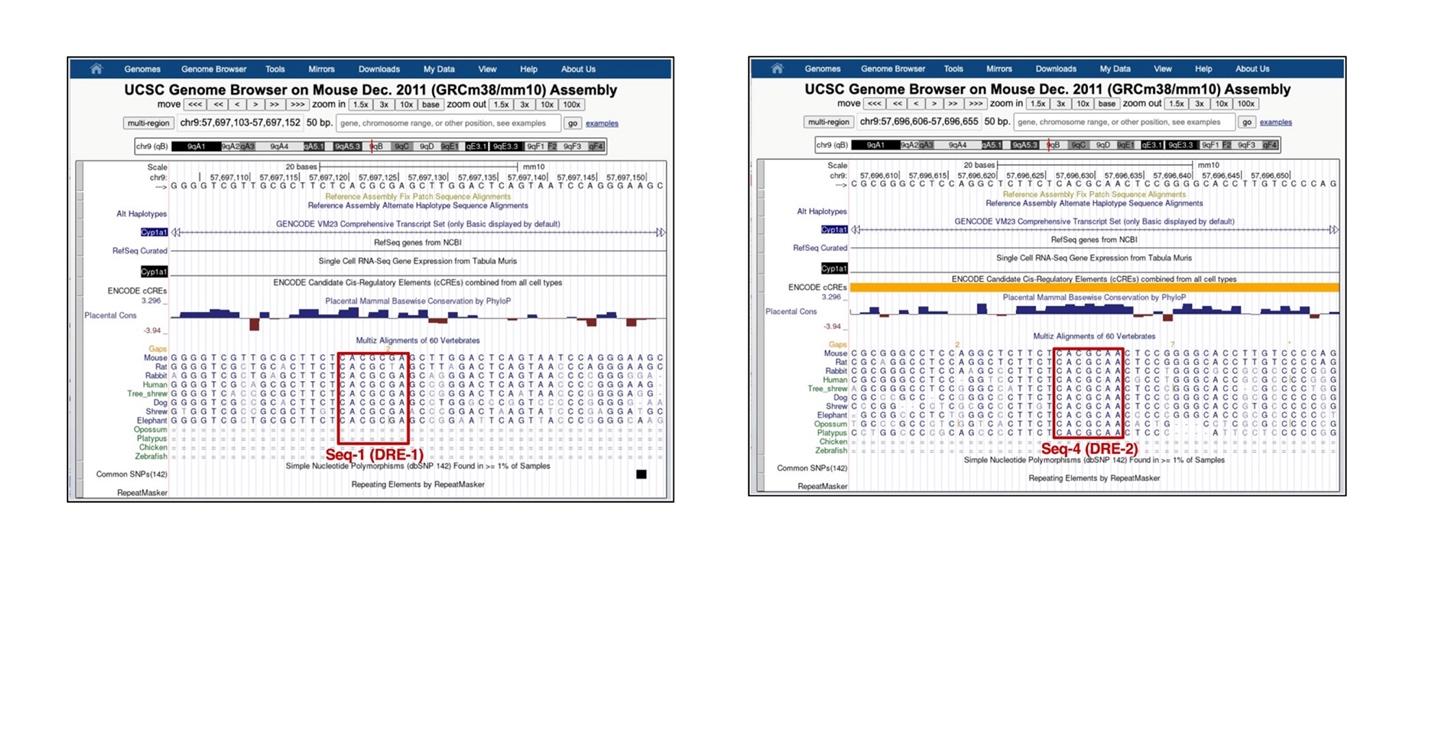


**Supplemental Fig. S2.**

Dioxin-responsive elements. Two AHR/dioxin-responsive elements (DRE-1 and 2) conserved between human and mouse provided by the UCSC database.

**
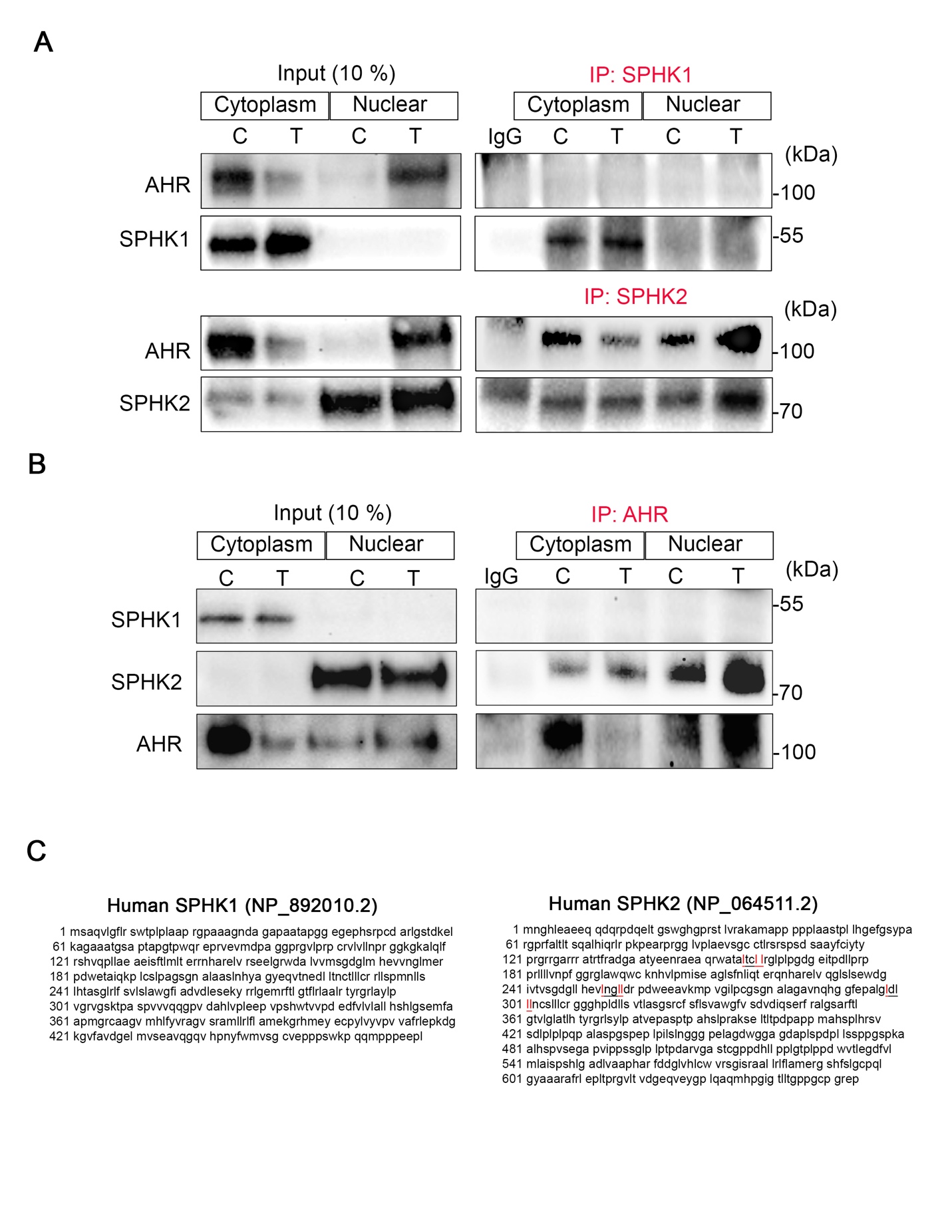
**

**Supplemental Fig.S3.**

Endogenous SPHK2 but not SPHK1 interacts with AHR in HeLa cells. HeLa cells treated with or without TCDF (0.1 µM for 30 m) were subjected to cytoplasmic/nuclear extract fractionation and immunoprecipitated using (**A**) SPHK1 or SPHK2 antibodies. The presence of the AHR in each SPHK1-IP or SPHK2-IP was assessed. C, Control; T, TCDF treated samples. (**B**) AHR antibody. The presence of SPHK1 or SPHK2 in AHR-IP was assessed. C, Control; T, TCDF treated samples. (**C**) The comparison of amino sequence between human SPHK1 and SPHK2. Red “l” and underline indicates the position of LXXLL sequence.


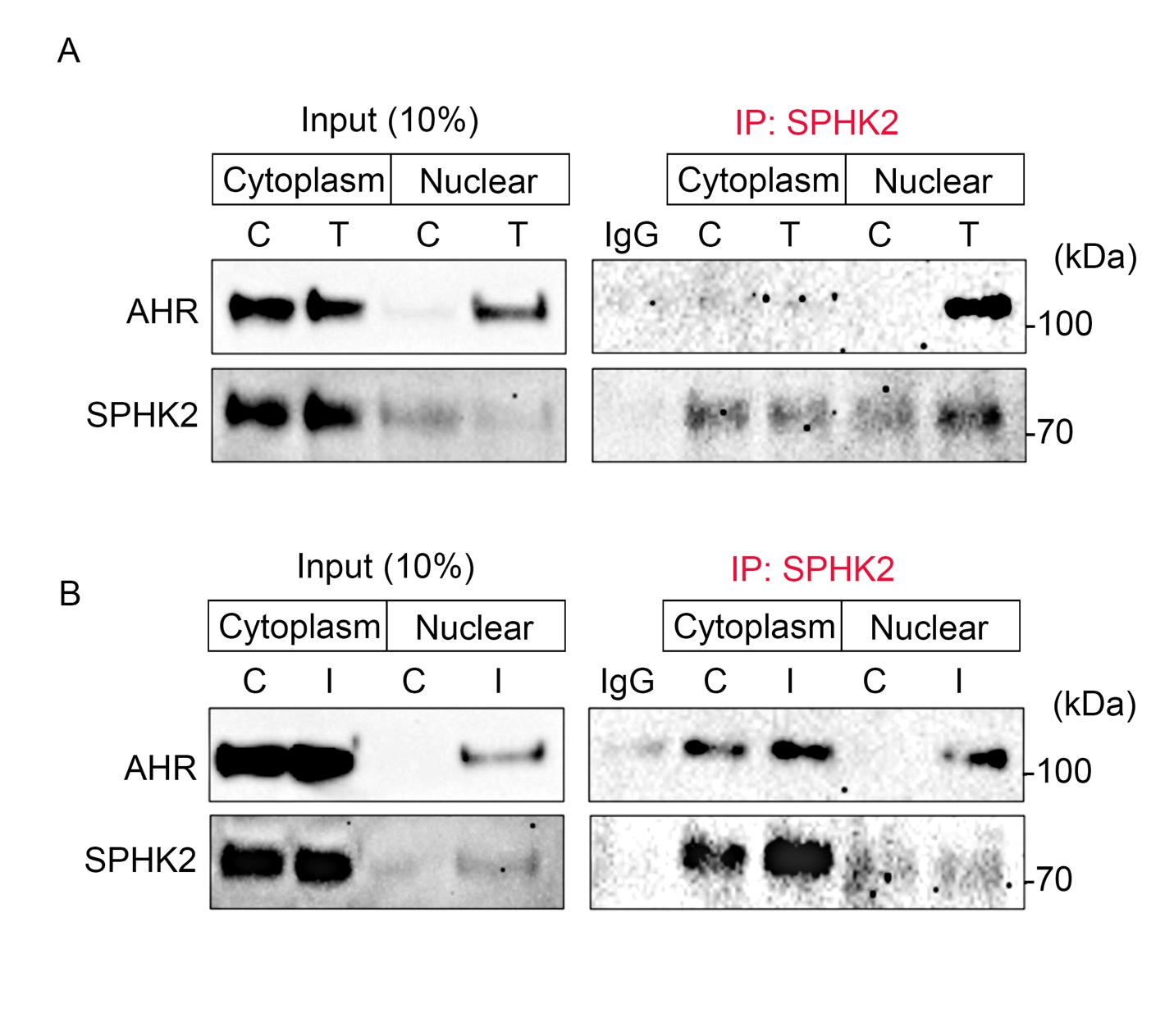


**Supplemental Fig. S4.**

Endogenous AHR and SPHK2 interact in Huh7 cells.

(**A**) Huh7 cells treated with or without TCDF (0.1 µM) were subjected to cytoplasmic/nuclear extract fractionation and immunoprecipitated using anti-SPHK2 antibody. Western blot analysis was performed with AHR and SPHK2 antibodies. C, Control; T, TCDF treated sample, respectively. (**B**) HeLa cells treated with or without ICZ (100 nM) and subjected to cytoplasmic/nuclear extract fractionation and subsequently immunoprecipitated using SPHK2 antibody. C, Control; I, ICZ treated.

**
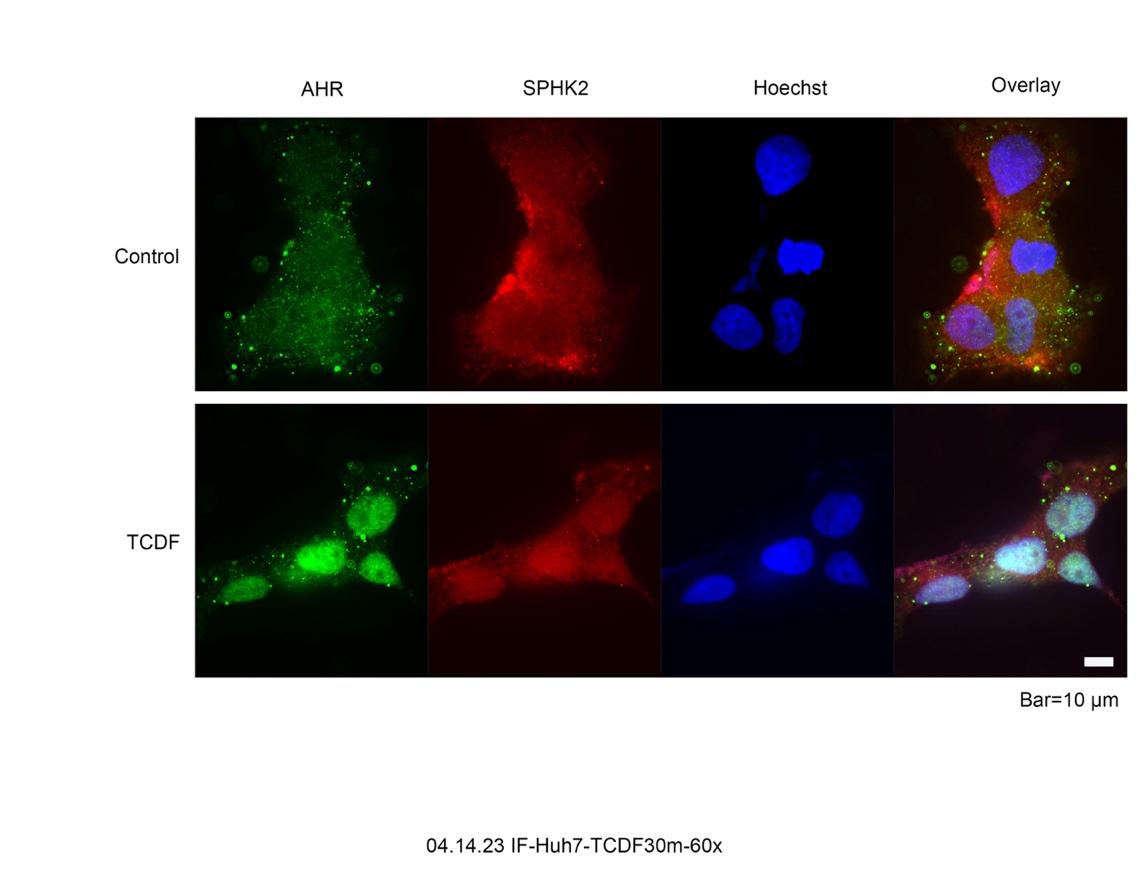
**

**Supplement Fig. S5.**

Colocalization of endogenous AHR and SPHK2 in TCDF treated Huh7 cells.

Immunofluorescent double staining using AHR and SPHK2 antibodies in Huh7 cells. The nucleus was counterstained with Hoechst 33342. Data are representative images from three independent experiments. Bar is 10 µm.


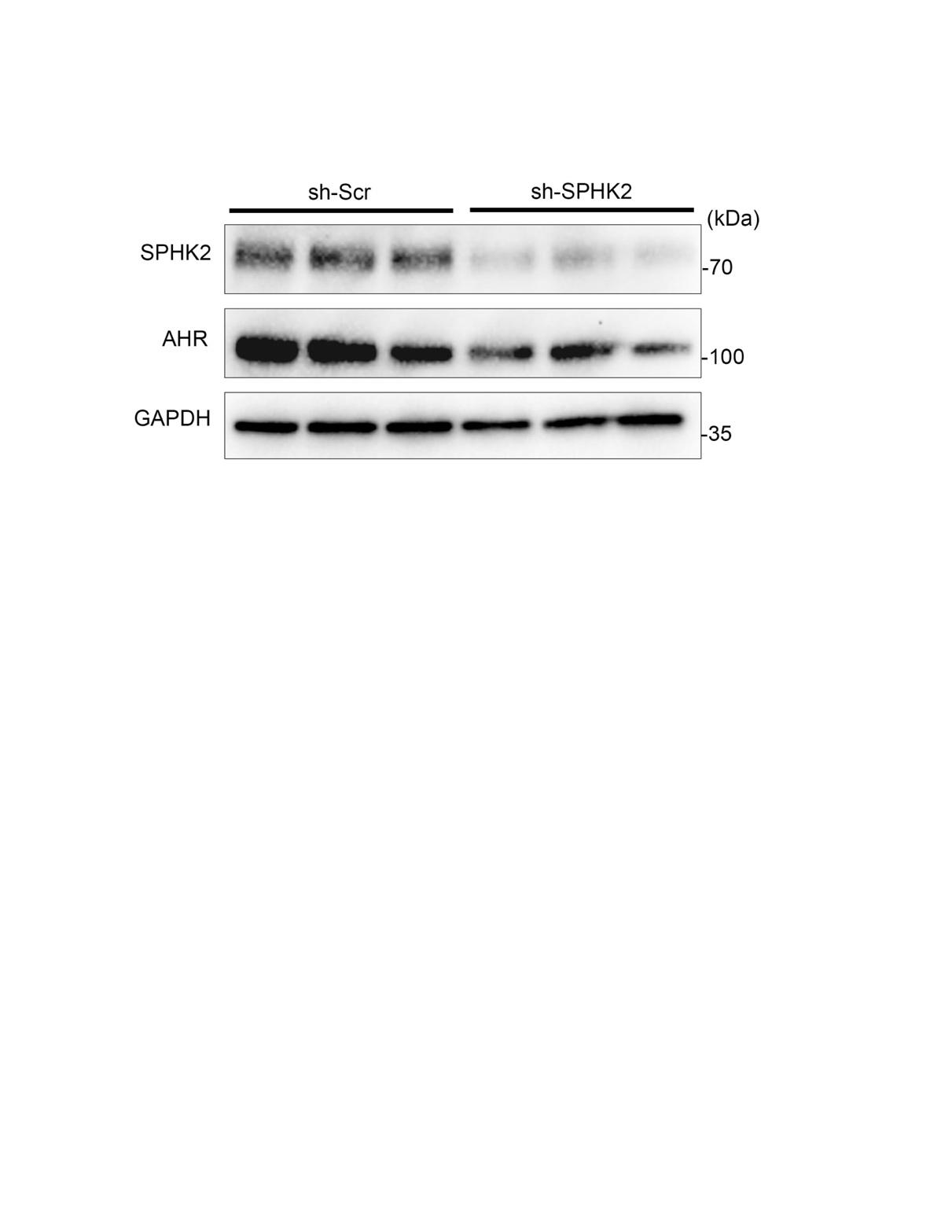


**Supplemental Fig. S6.**

Knockdown of SPHK2 downregulates AHR expression in Huh7 cells.

shRNA for SPHK2 transfected into Huh7 cells reduced AHR protein expression as confirmed by western blotting.


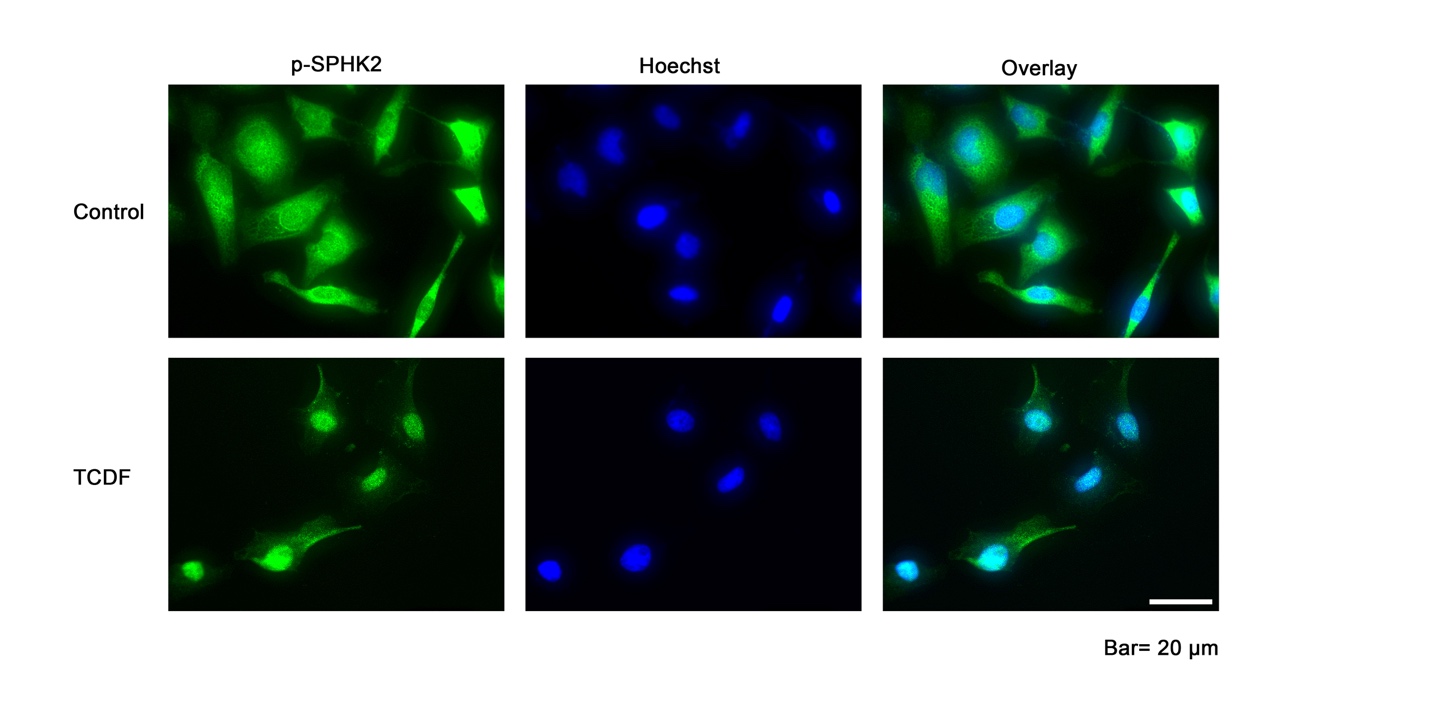


**Supplemental Fig. S7.**

Lower magnification of immunofluorescent images of p-SPHK2 expressions in HeLa cells with/without TCDF (0.1 µM) for 30 m. Nucleus were counter stained with Hoechst 33342. Bar= 20 µm.


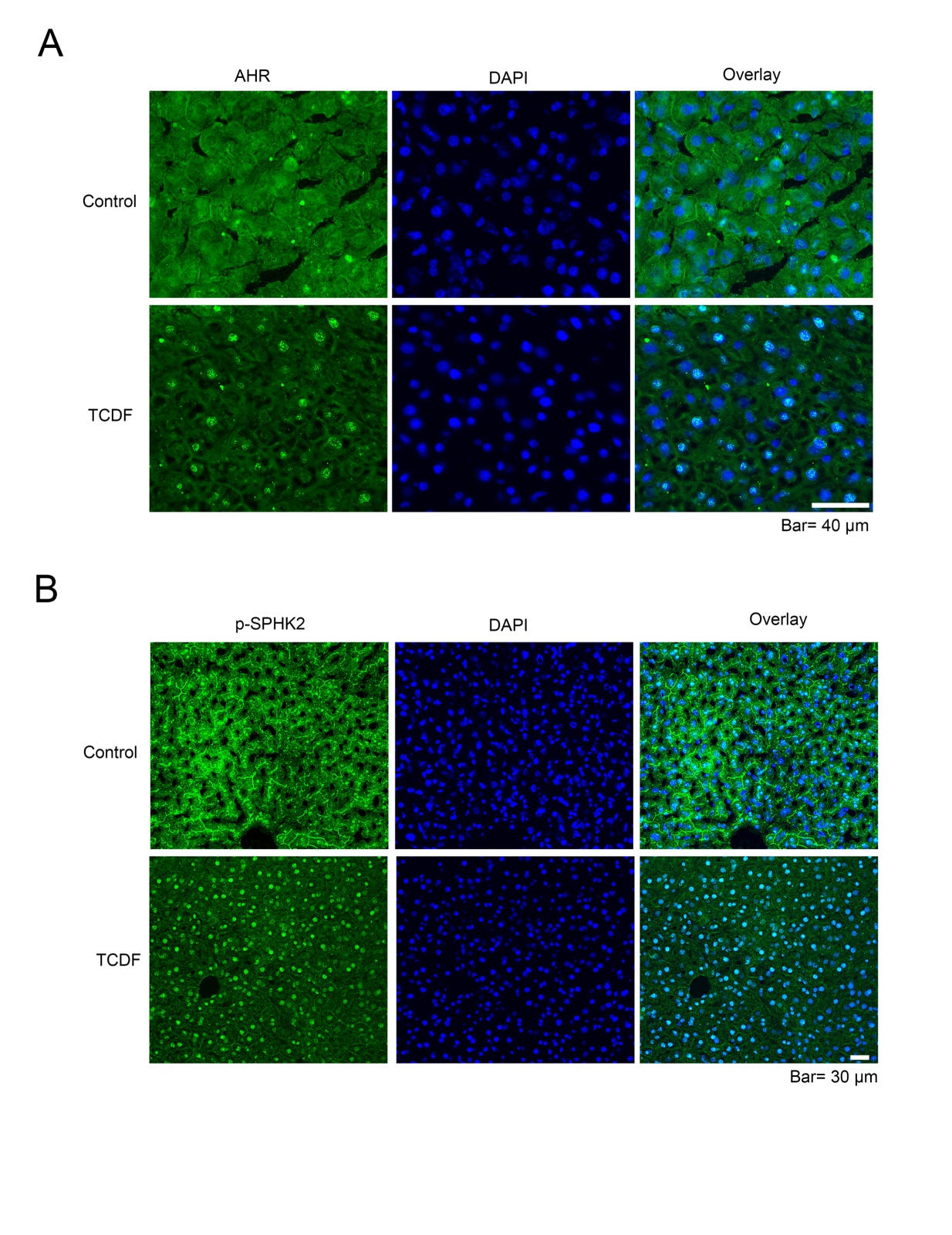


**Supplemental Fig. S8.**

TCDF promotes nuclear p-SPHK2 in mouse liver. (**A**) Immunofluorescent staining of AHR in mouse liver tissue with or without TCDF treatment (24 µg/kg). Bar=40 µm. (**B**) Immunofluorescent staining of p-SPHK2 expression in mouse liver tissue with or without TCDF treatment. The nucleus was counterstained with DAPI. Bars= 30 µm. (n=3)


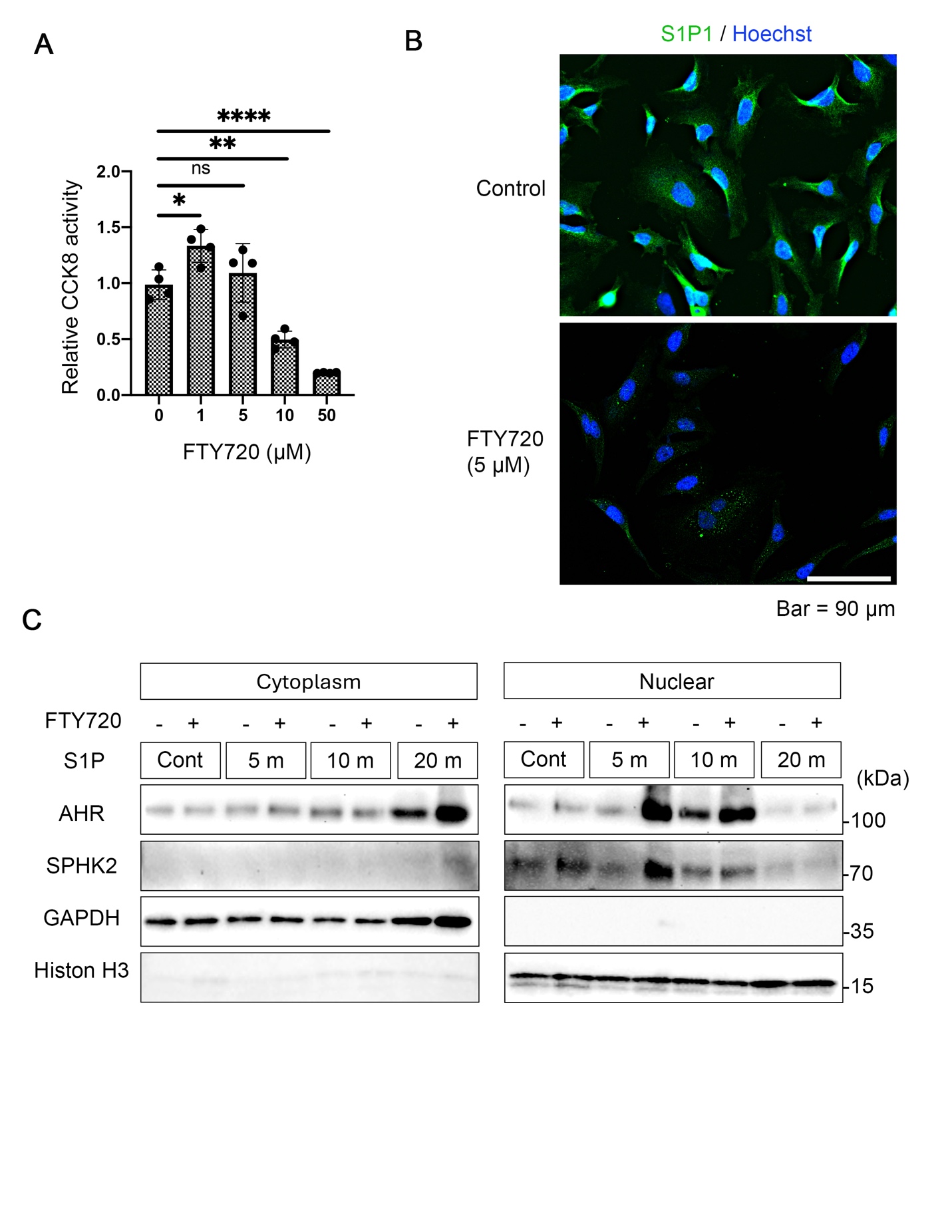


**Supplemental Fig. S9.** FTY720 quickly and transiently enhanced AHR/SPHK2 expression in the nucleus. **(A)** CCK8 assay results after 24 h incubation with different doses of FTY720 (µM). (**B**) Immunofluorescence images of HeLa cells for S1P1 after pretreated with FTY720 (5 µM) for 1 h. Bar= 90 µm. (**C**) Western blot analysis of HeLa cells after S1P treatment for 5, 10, 20 min. (n=3). FTY720 “+” samples were pre-treated with FTY720 (5 µM) for 1 h before S1P administration. Cells were fractionated into cytoplasmic and nuclear extracts and subjected to western blotting analysis with the indicated antibodies. (n=3).


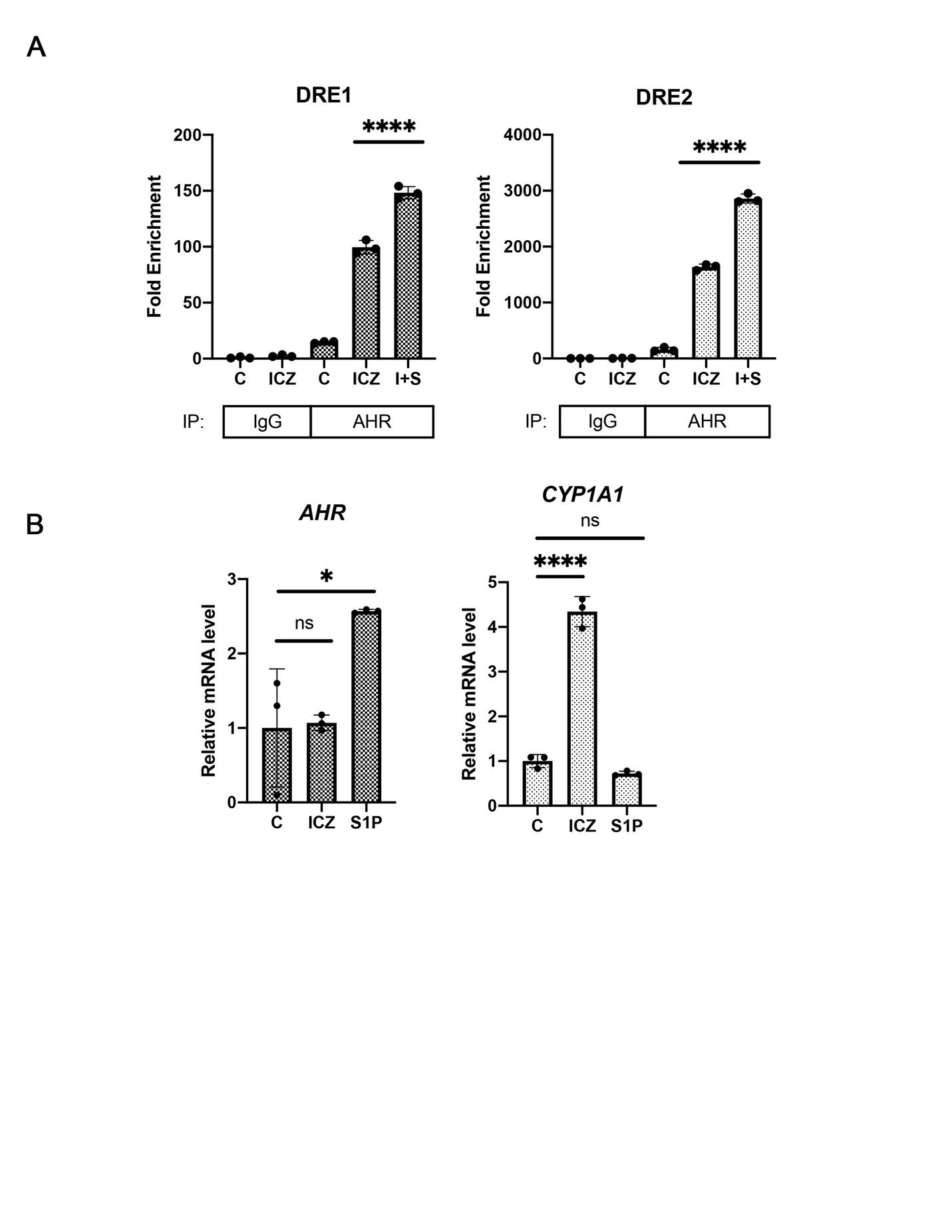


**Supplemental Fig. S10.**

ICZ+S1P enhances AHR recruitment on DREs in Huh7 cells.

(**A**) ChIP-qPCR analysis for the presence of the AHR in the two DRE in the *CYP1A1* promoter in Huh7 cells treated with or without ICZ (0.1 µM), S1P (1 µM) for 5 min. Normal rabbit IgG was used for the negative control. C, control; I, ICZ; S, S1P. **(B)** qRT-PCR analysis for AHR, CYP1A1 induction after ICZ or S1P treatment for 2 h in Huh7 cells. All data are representative of independent three experiments. ****p<0.0001, ***p<0.001, **p<0.01. *p<0.05.


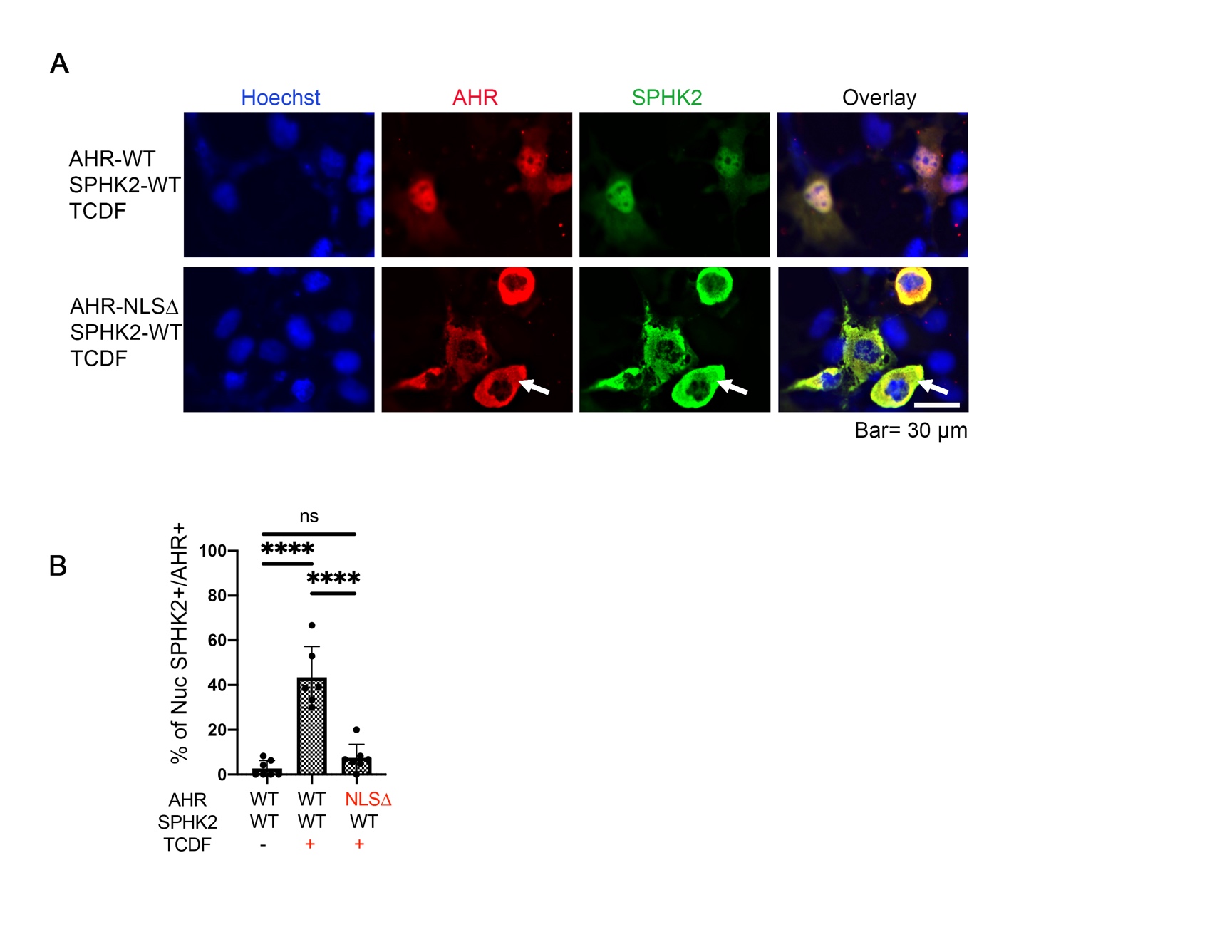


**Supplemental Fig.S11.**

(**A**) Immunofluorescence images of Cos-1 cells transfected with AHR-WT, SPHK2-WT, AHR-NLS deletion (NLSΔ) plasmid. After TCDF treatment (0.1 µM, 30 m), cells were fixed and processed for immunofluorescence staining using each antibody. Arrows indicate cytoplasmic co-localization of AHR and SPHK2. The nucleus was counterstained with Hoechst 33342. Bar= 30 µm. (**B**) Quantitative analysis of percentage of SPHK2+ nuclear staining per AHR + cells in randomly selected independent fields. n>100 (fields n>6).


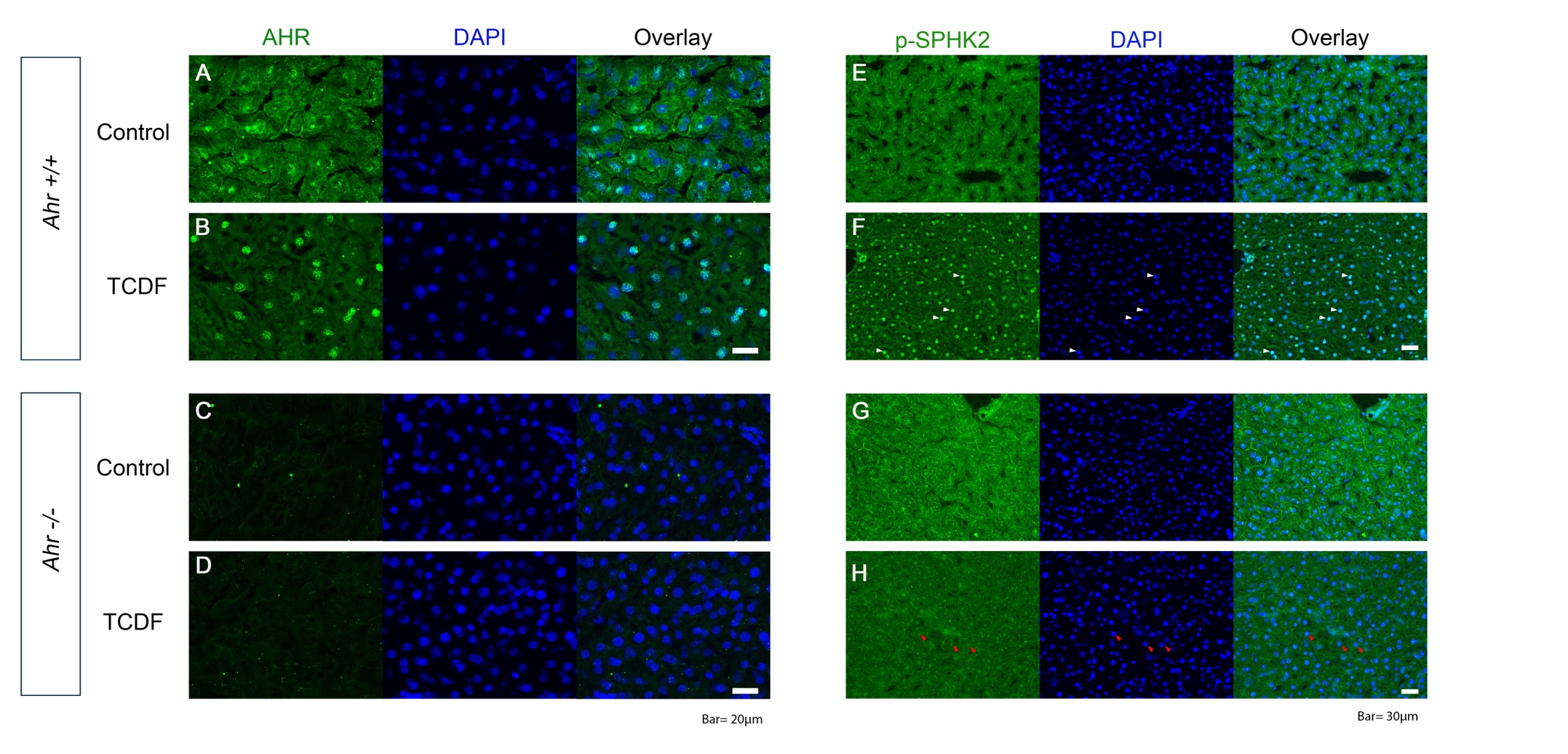


**Supplemental Fig. S12.**

Immunofluorescence images of WT (*Ahr^+/+^*, **A, B, E, F** ) or *Ahr*-null (*Ahr ^-/^*^-^, **C, D, G, H**) mouse liver treated with (**B, D, F, H**) or without (**A, C, E, G**) dietary TCDF (24 µg/kg for 24 h). Tissues were processed for frozen sectioning and stained using AHR (**A-D**) or p-SPHK2 antibody (**E-H**). White arrowheads indicate enhanced p-SPHK2 expressions in the nucleus. Red arrowheads indicate minimum expressions of p-SPHK2 in nucleus. The nucleus was counterstained with DAPI. Bar=20 µm (B, D), 30 µm (F, H), respectively (n=3).

**Supplemental Table S1.** Six putative AHR binding sequences (Seq-1, -2, -3, -4, -5 and-6) in the mouse *Cyp1a1* promoter provided by the JASPR database.

**
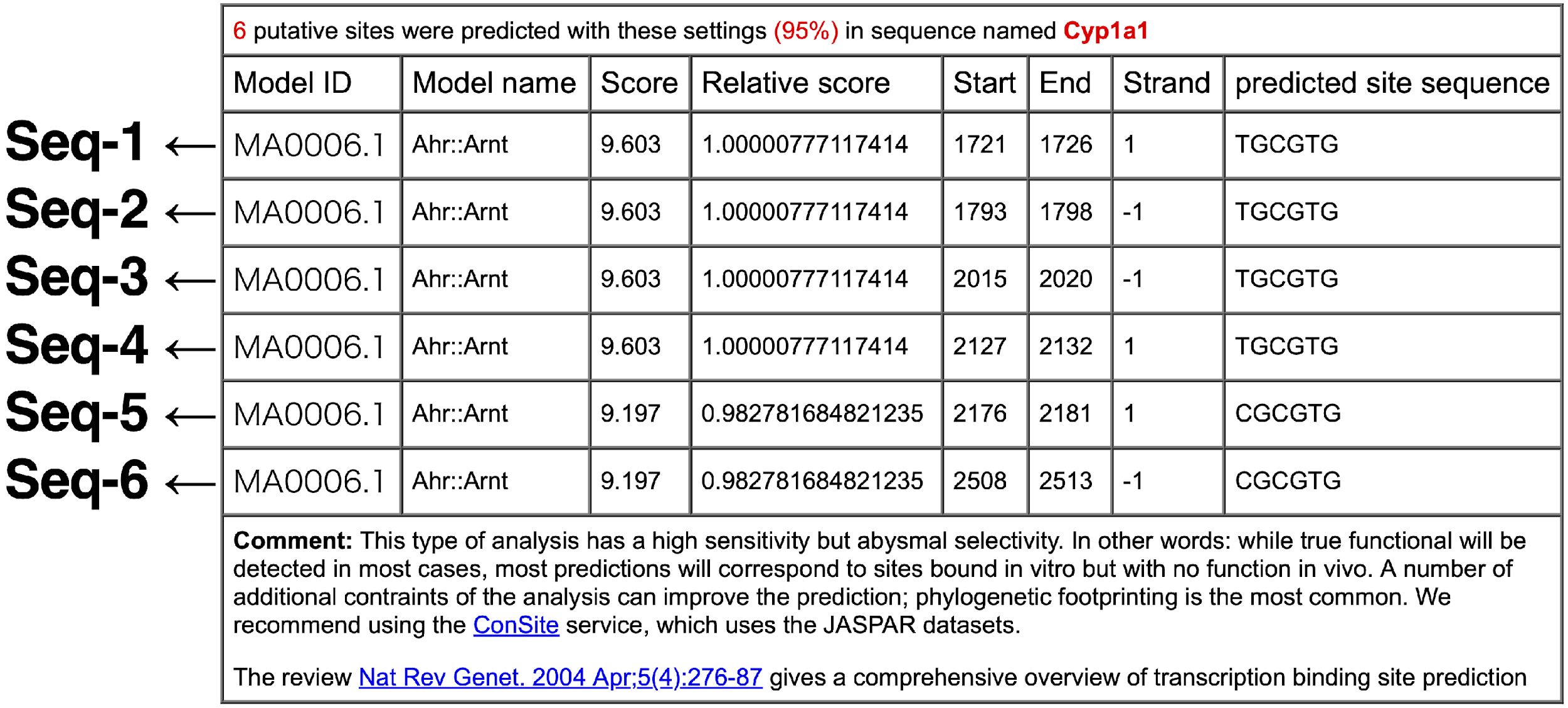
**

**Supplemental Table S2.** Key resource table

| Reagent type (species) or resource | Designation | Source or reference | Identifiers | Additional information |
| --- | --- | --- | --- | --- |
| Antibody | Rabbit monoclonal Anti-AHR | Cell Signaling technology | #83200, RRID:AB_2800011 | IP: 1:100, IF: 1:100, WB: 1:1000, ChIP: 1:100 |
| Antibody | Mouse monoclonal Anti-AHR | Invitrogen | # 14-9854-82, RRID:AB_2865478 | IF: 1:100 |
| Antibody | Goat polyclonal Anti-AHR | Novus Biologicals | #NB100-128, PRID: AB_10001635 | IF: 1:300 |
| Antibody | Rabbit Polyclonal Anti-HIF-1b/ARNT | Cell Signaling technology | #5537, RRID:AB_10694232 | WB: 1:1000 |
| Antibody | Rabbit polyclonal Anti-SPHK2 | Cell Signaling technology | #32346, RRID:AB_2799021 | WB: 1:1000, IP: 1:100, 1F: 1:100, ChIP: 1:100 |
| Antibody | Rabbit polyclonal Anti-Phospho-Sphk2 (T614) | Bosterbio | #A01382T614 | WB: 1:1000, IF: 1:100 |
| Antibody | Rabbit polyclonal Anti-SPHK1 | Cell Signaling technology | #12071S, RRID:AB_2797815 | WB: 1:1000 |
| Antibody | Rabbit polyclonal Anti-B-Actin (13E5) | Cell Signaling technology | #4970, RRID:AB_2223172 | WB: 1:1000 |
| Antibody | Rabbit polyclonal Anti-S1P1 | Invitrogen | # **PA1-1040**, RRID:AB_2184729 | IF: 1:100 |
| Antibody | Rabbit monoclonal Anti-GAPDH (D16H11) | Cell Signaling technology | #5174, RRID:AB_10622025 | WB: 1:1000 |
| Antibody | Rabbit monoclonal Anti-Histon H3 (D1H2) | Cell Signaling technology | #4499, RRID:AB_10544537 | WB: 1:1000 |
| Antibody | Rabbit monoclonal Anti-HA-Tag | Cell Signaling technology | #3724, RRID:AB_1549585 | WB: 1:1000 |
| Antibody | Mouse monoclonal Anti-rabbit IgG (Conformation Specific) mAb (HRP Conjugate) | Cell Signaling technology | #5127, RRID:AB_1549606 | WB: 1:2000 |
| Antibody | Rabbit monoclonal Anti-Mouse IgG (Light Chain Specific) mAb (HRP Conjugate) | Cell Signaling technology | #58802, RRID:AB_2799549 | WB: 1:2000 |
| Antibody | Donkey polyclonal Anti-Goat IgG (H+L) Cross-Adsorbed Secondary Antibody, Alexa Fluor™ 488 | Invitrogen | #A-11055, RRID:AB_2534102 | IF: 1:300 |
| Antibody | Donkey polyclonal Anti-Rabbit IgG (H+L) Highly Cross-Adsorbed Secondary Antibody, Alexa Fluor™ 594 | Invitrogen | #A21207, RRID:AB_141637 | IF: 1:300 |
| Antibody | Donkey anti-Mouse IgG (H+L) Highly Cross-Adsorbed Secondary Antibody, Alexa Fluor™ 488 | Invitrogen | #A21202, RRID:AB_141607 | IF: 1:300 |
| Antibody | Goat anti-Rabbit IgG (H+L) Cross-Adsorbed Secondary Antibody, Alexa Fluor™ 488 | Invitrogen | #A11008, RRID:AB_143165 | IF: 1:300 |
| Chemical compounds | Hoechst33342 | Thermo Scientific | #62249 |  |
| Chemical compounds | DAPI [4'',6-Diamidino-2-phenylindole, dihydrochloride] | ANASPEC | # AS-83210 |  |
| Chemical compounds | SPHK2 inhibitor ABC294640 | Echelon Biosciences | #B-0025 |  |
| Chemical compounds | S1P-immobilized agarose beads | Echelon Biosciences | #S-2000 |  |
| Chemical compounds | Control beads | Echelon Biosciences | #P-B000 |  |
| Chemical compounds | Protein G Magnetic beads | BioLabs | #S1430S |  |
| Chemical compounds | S1P bioactive lipid | SIGMA | #73914 |  |
| Chemical compounds | 2,3,7,8-tetrachlorodibenzofuran (TCDF) | Cambridge Isotope Laboratories | #EF-903-C |  |
| Chemical compounds | Indolo[3,2-b] carbazole (ICZ) | Matrix Scientific | #024785 |  |
| Chemical compounds | Gibco^TM^ DMEM, high glucose | Thermo Fisher Scientific | #11965-092 |  |
| Chemical compounds | Fetal bovine serum (FBS, heat-inactivated) | R&D Systems | #S11150H |  |
| Chemical compounds | Penicillin/streptomycin | Gibco | #15140122 |  |
| Chemical compounds | DMEM, high glucose, GlutaMAX™ Supplement, pyruvate | Thermo Fisher Scientific | #10569010 |  |
| Chemical compounds | cOmplete^™^, Mini, EDTA-free Protease Inhibitor Cocktail | SIGMA | #11836170001 |  |
| Chemical compounds | Phenylmethylsulphonyl fluoride (PMSF) | SIGMA | #41216525 |  |
| Chemical compounds | 4–20% Criterion™ TGX Precast Midi Protein Gel | BioRad | #5671094 |  |
| Chemical compounds | Non-fat dry milk | Santacruz | #sc-2325 |  |
| Chemical compounds | TRizol ^TM^ Reagent | Thermo Fisher Scientific | #15596026 |  |
| Chemical compounds | Formaldehyde solution | AMRESCO | #0493 |  |
| Chemical compounds | MeOH | SIGMA | #MX0485-3 |  |
| Chemical compounds | Donkey serum | Jackson | #07130118 |  |
| Chemical compounds | BSA, Fraction V | Caibiochem | #2960 |  |
| Chemical compounds | Trypsin-EDTA (0.25%) | Thermo Fisher Scientific | #25200056 |  |
| Chemical compounds | PBS (Gibco™, pH 7.4) | Fisher Scientific | #10-010-072 |  |
| Chemical compounds | MOPS/EDTA Buffer | Calbiochem | #475916 |  |
| Chemical compounds | EDTA | SIGMA | #E4884 |  |
| Chemical compounds | Sodium azide (NaN3) | SIGMA | #S2002 |  |
| Chemical compounds | Glycerol | VWR | #BDH1172 |  |
| Chemical compounds | Sodium molybdate | SIGMA | #331058 |  |
| Chemical compounds | Tris base | VWR | #0497 |  |
| Chemical compounds | Mercaptoethanol | BioRad | #1610710 |  |
| Chemical compounds | SDS | SIGMA | #7910 |  |
| Chemical compounds | Bromophenol blue | SIGMA | #B5525 |  |
| Chemical compounds | Tween20 | SIGMA | #P1379 |  |
| Chemical compounds | DMSO | SIGMA | #D8418 |  |
| Chemical compounds | Transgenic Dough Diet™ - Sterile | Bio-Serv | #S3472 |  |
| Commercial assay kit | Pierce BCA Protein Assay kit | Thermo Fisher Scientific | #23225 |  |
| Commercial assay kit | Lipofectamine™ 3000 Transfection Reagent | Thermo Fisher Scientific | # L3000015 |  |
| Commercial assay kit | Cell Counting Kit-8 (CCK8) assay | Dojindo | #CK04-11 |  |
| Commercial assay kit | SimpleChIP® Enzymatic Chromatin IP kit (Magnetic Beads) | Cell Signaling Technology | #9003 |  |
| Commercial assay kit | SuperSignal™ West Dura | Thermo Fisher Scientific | #34075 |  |
| Commercial assay kit | qScript cDNA Supermix | Quantabio | #95048 |  |
| Commercial assay kit | PowerUp SYBR Green Real-Time PCR Master Mix | Applied Biosystems | #A25742 |  |
| Commercial assay kit | 4–20% Criterion™ TGX Precast Midi Protein Gel | BioRad | #5671094 |  |
| Commercial assay kit | Trans-Blot Turbo Transfer System Transfer Pack | BioRad | #1704157EDU |  |
| Commercial assay kit | Q5^®^ Site-Directed Mutagenesis Kit | NEB | #E0554S |  |
| Cell line | HeLa cells (Human) | ATCC | N/A |  |
| Cell line | Huh7 cells (Human) | ATCC | N/A |  |
| Cell Line | COS-1 cells (Monkey) | ATCC | N/A |  |
| Software, algorithm | GraphPad Prism (version 7) | GraphPad Software, | https://www.graphpad.com |  |
| Software, algorithm | Zen Black (version 3.1) | Zeiss | https://www.zeiss.com/microscopy/int/products/microscope-software/zen-lite |  |
| Software, algorithm | Adobe Photoshop CC | Adobe | https://www.adobe.com/uk/products/photoshop |  |
| Software, algorithm | Fiji2-imageJ | Fiji | https://imagej.net/software/fiji/downloads |  |
| Recombinant DNA | pPM-C-SPHK2-HA | Applied Biological Materials | #453130210500 |  |
| Recombinant DNA | sh-SPHK2 construct | ORIGENE | #TG301412 |  |
| Recombinant DNA | pCI-hAHR-FLAG (DeltaNLS) |  | Biochemistry. 2004; 43:700-709. |  |

Oligonucleotides

| Amplicon | F primer (5'-->3') | R primer (5'-->3') | Used for | Reference |
| --- | --- | --- | --- | --- |
| *hSPHK2* | GAGCCTGAGTGAGTGGGATG | CAGTCAGGGCGATCTAGGAG | qRT-PCR | This study |
| *hAHR* | ATTGTGCCGAGTCCCATATC | TGCATTAGACTGGACCCAAG | qRT-PCR | This study |
| *hCYP1A1* | GGTCAAGGAGCACTACAAAACC | TGGACATTGGCGTTCTCAT | qRT-PCR | This study |
| *hGAPDH* | TGTGGTCATGAGTCCTTCCACGA | AGCCTCAAGATCATCAGCAATGCC | qRT-PCR | This study |
| DRE-1 | AGGCGCGAACCTCAGCTAGT | GCTACAGCCTACCAGGACTCG | Chip-qPCR | This study |
| DRE-2 | GTCCTTCTCACGCAACG | TTGGCAGAGCACAGAAATCC | Chip-qPCR | This study |
| shSPHK2-A | GAATGGACACCTTGAAGCAGAGGAGCAGC |  | shRNA | This study |
| shSPHK2-B | GCATCGTTCTGTGTCTGACCTGCCTCTTC |  | shRNA | This study |
| shSPHK2-C | GTCCTTCAACCTCATCCAGACAGAACGAC |  | shRNA | This study |
| shSPHK2-D | CCACTGCTGTCTTCACCTCCTGGCTCTCC |  | shRNA | This study |

**Supplemental Table S3.**

Sphingolipid synthesis proteins and LXXLL motifs.

Representative human and mouse sphingolipid synthesis proteins possess the LXXLL motifs. Hydrophobic residue (isoleucine, I; valine, V; leucine, L; phenylalanine, F; cysteine, C; methionine, M; alanine, A; and, tryptophan, W) are shown in yellow. Basic amino acid (arginine, R; histidine, H; and lysine, K) are shown in green. aa, amino acid.


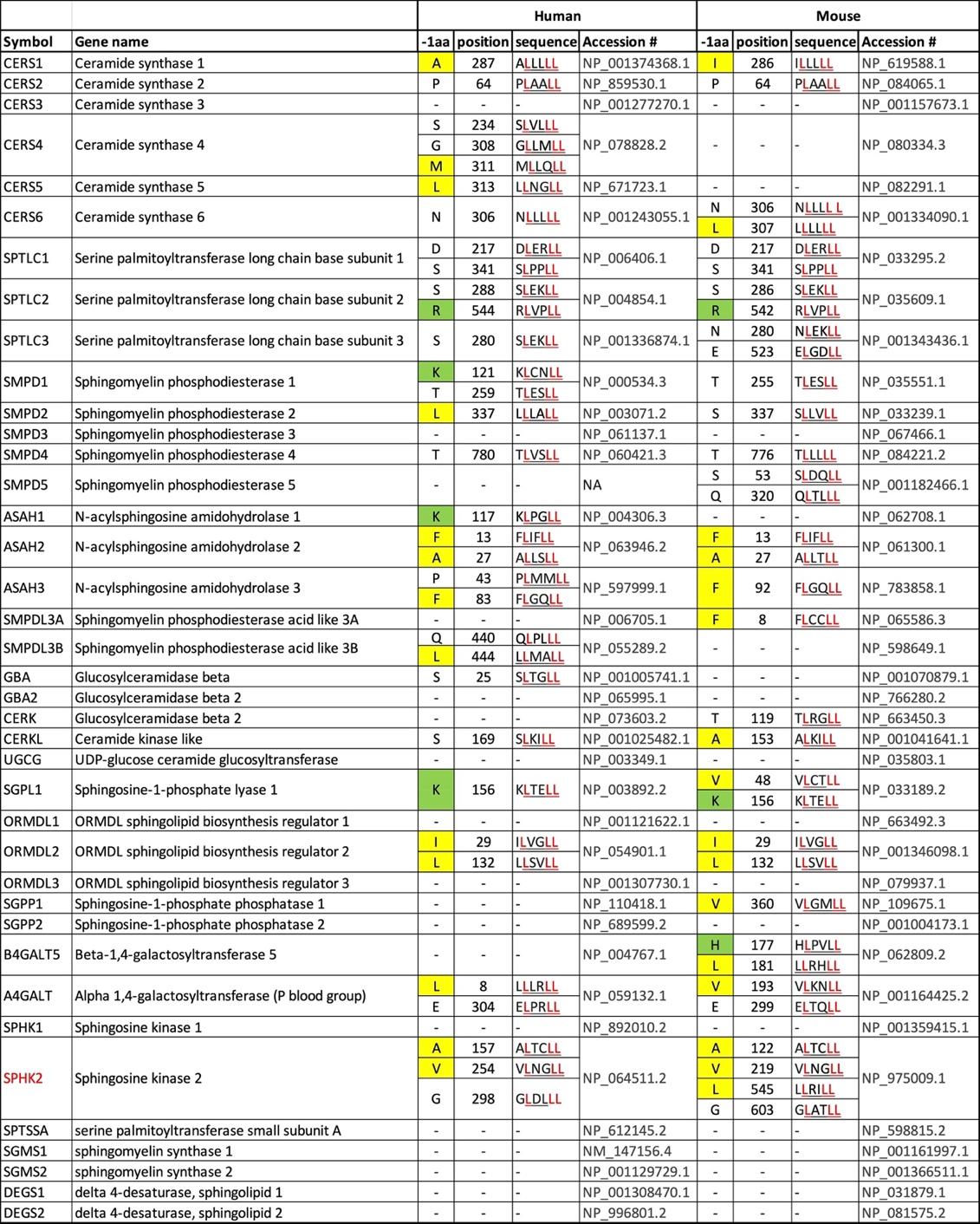

Supplement: Supplementary file 1 — Supporting Information [file ADVS-11-2400794-s001.docx]
